# Supplementary material for: Inequality of gender, age and disabilities due to leprosy and trends in a hyperendemic metropolis: Evidence from an eleven-year time series study in Central-West Brazil
Source: PLoS Negl Trop Dis. 2021 Nov 16;15(11):e0009941. doi: 10.1371/journal.pntd.0009941 (PMC8631739; doi:10.1371/journal.pntd.0009941)
Supplement: S1 File — (DOCX) [file pntd.0009941.s001.docx]

STROBE Statement—checklist of items that should be included in reports of observational studies

|  | Item No. | Recommendation | Page  No. | Relevant text from manuscript |
| --- | --- | --- | --- | --- |
| **Title and abstract** | 1 | (*a*) Indicate the study’s design with a commonly used term in the title or the abstract | Page 1 | Inequality of gender, age and disabilities due to leprosy and trends in a hyperendemic metropolis: Evidence from an eleven-year time series study in Central-West Brazil |
|  |  | (*b*) Provide in the abstract an informative and balanced summary of what was done and what was found | Page 2 | The present study aimed to investigate the epidemiological situation of leprosy, its behavior over eleven years, and whether there were trends, both in the detection of the disease and in disabilities, analyzing disparities and/or differences regarding gender and age in a hyperendemic metropolis in the Central-West region of Brazil. Ecological time series study, conducted in Cuiabá, capital of the state of Mato Grosso. The population was composed of patients diagnosed with leprosy between the years 2008 and 2018. Descriptive statistics were applied, with the calculation of absolute frequencies (*n*) and relative frequencies (%).The time series of leprosy cases was used, stratifying it according to gender (male and female), Grade of disability (G0D, G1D, G2D and not evaluated) and age. The calendar adjustment technique was applied, taking into account the number of days of each month in the calculation. For modeling the trends, the Seasonal-Trend decomposition procedure based on Loess (STL) was used. We identified 9,739 diagnosed cases, in which 58.37% were male and 87.55% aged between 15 and 59 years. Regarding detection according to gender, there was a decrease among women and an increase in men. There was a trend towards an increase in disabilities in both genders. The study advances the knowledge by evidencing a increasing trend of leprosy cases among women, as well as increases in disabilities in both genders, which may be related to the delay in diagnosis. There was also an increasing number of cases that were not assessed for disability at the time of diagnosis, which denotes the quality of the services. |
| Introduction | | | |  |
| Background/rationale | 2 | Explain the scientific background and rationale for the investigation being reported | Pages 3 - 5 | Leprosy is a chronic infectious disease caused by the bacillus *Mycobacterium leprae*, which affects Schwann cells, causing their destruction, affecting the skin and resulting in severe neuropathies, which lead to physical disabilities. It is believed that its transmission takes place through droplets, however, skin contact or other means cannot be excluded [1]. In the previous 30 years, the World Health Organization (WHO) has been seeking measures to eliminate leprosy, and although its indicators have been decreasing over the years, the goal of elimination (prevalence <1 case per 10,000 inhabitants) has not yet been reached [2,3] and currently seems to be more distant than imagined.  In 2016, according to WHO data, 143 countries had a detection rate of 2.9 cases per 100,000 inhabitants and, in the same year, Brazil had a detection rate of 12.2 cases per 100,000 inhabitants, being classified as a high disease burden country [4]. It is the country with the second highest number of leprosy occurrences, only behind India, and its contribution to the burden of leprosy in the Americas is significant, accounting for 92.3% of cases in the continent [5].  According to the “Global Leprosy Strategy 2016−2020: Accelerating towards a leprosy-free world” [1], the main actions to control the disease should be directed toward early detection, reduction in the number of cases and reduction of cases with deformities, mainly in children. In addition, there should be a reduction in the time of diagnosis, preventing cases with grade of disability. It is also expected that the services will systematically assess their diagnosed cases regarding disabilities, in a longitudinal manner throughout the treatment, thus avoiding more severe sequelae. The Plan reinforces the prevention and management of physical disabilities, by interprofessional teams, the search for contacts, careful and planned monitoring of cases already diagnosed and the reduction of stigma, a major complication in seeking services and treatment adherence [5-7].  Brazil is a country of continental proportions, with an extensive land border, neighboring 10 of the 12 other countries in South America, which makes leprosy control a major challenge. It is divided into five macro-regions, among which the Central-West is one of the most problematic regions in terms of the burden of the disease. A study carried out in the region showed that in the trienniums of 2001 - 2003 and 2010 - 2012 there was a reduction in the disease, however, there are geographical areas that leprosy control has not advanced and are far from elimination [8]. The official reports from Brazil indicate the reduction of leprosy in general, however, there is evidence that there is gender inequity in access to health services, which may have implications for the disease [9]. Another issue refers to age, as there is evidence that the population aging process is changing the profile of the leprosy morbidity profile [10], given the number of people that are falling ill in a context of poverty and inequality, with older adults having more difficulty in accessing health services, and also tending to have more unfavorable prognosis. This needs to be better addressed from the perspective of health surveillance.  Also regarding the inequality related to age, it is known that when there is a delay in diagnosis, children that had contact with index cases also become ill [11], which is an important gap to be filled. Accordingly, the elimination of leprosy involves comprehending the determinants, according to a gender and age equity perspective. It is also understood that gender and age inequity should not be analyzed only from the perspective of confirmed detection and/or diagnosis, but also in terms of disability, as the WHO recognizes the grade of disabilitie indicator as the most sensitive measure of the real leprosy situation in a community, expressing, therefore, the fragility of the services in detecting the disease early [1].  There are hypotheses that there are gender differences, and that older adults and children are more severely affected by the disease, with regard to disabilities, and that this will continue in the coming years. These aspects need to be studied, in order to define public policies and plan strategic actions in priority areas, as well as to advance equity. There are several tools that could be used to test these hypotheses, among them, one of the more sensitive is the time series. Its use is justified in the field of public health, as it can show the behavior of the disease in vulnerable groups and verify how much success has been achieved in terms of the goal of elimination and reduced of injustices and/or inequity. Although time series studies have been exhaustively applied to identify the dynamics of COVID-19 and even make predictions, supporting decisions regarding lockdowns and or social isolation [12,13], there are few studies with leprosy with that have this intentionality [14]. |
| Objectives | 3 | State specific objectives, including any prespecified hypotheses | Page 5 | Based on the above, this study aimed to investigate the epidemiological situation of leprosy and trends in the detection of cases and disabilities, and to evidence disparities and/or differences regarding gender and age in a hyperendemic metropolis in Central-West Brazil. |
| Methods | | | |  |
| Study design | 4 | Present key elements of study design early in the paper | Page 5 | This ecological time series study [15] was carried out in Cuiabá, capital of the state of Mato Grosso, located in the Central-West region of the country (Fig 1). |
| Setting | 5 | Describe the setting, locations, and relevant dates, including periods of recruitment, exposure, follow-up, and data collection | Page 7 | Leprosy cases registered in the Notifiable Disease Information System (SINAN) from 2008 to 2018 of residents of the city of Cuiabá were included. SINAN is the Brazilian information system responsible for recording and processing information on mandatory notifiable diseases such as leprosy throughout Brazil, providing bulletins and reports of morbidity and constituting one of the main surveillance systems in the country.  The selected variables were: date of notification of the case, gender (male, female), age, race/ skin color (white, black, yellow, mixed race and indigenous), education (no schooling, incomplete elementary education, complete elementary education, incomplete high school education, complete high school education, incomplete higher education and complete higher education), WHO operational classification (Paucibacillary, Multibacillary), clinical form based on Madrid classification (indeterminate, tuberculoid, borderline and lepromatous) and assessment of grade of disability in the diagnosis (Grade 0 disability [G0D], Grade 1 disability [G1D], Grade 2 disability [G2D] and not evaluated). |
| Participants | 6 | (*a*) *Cohort study*—Give the eligibility criteria, and the sources and methods of selection of participants. Describe methods of follow-up  *Case-control study*—Give the eligibility criteria, and the sources and methods of case ascertainment and control selection. Give the rationale for the choice of cases and controls  *Cross-sectional study*—Give the eligibility criteria, and the sources and methods of selection of participants | Not applicable | Not applicable, it is an ecological study. |
|  |  | (*b*) *Cohort study*—For matched studies, give matching criteria and number of exposed and unexposed  *Case-control study*—For matched studies, give matching criteria and the number of controls per case | Not applicable | Not applicable, it is an ecological study. |
| Variables | 7 | Clearly define all outcomes, exposures, predictors, potential confounders, and effect modifiers. Give diagnostic criteria, if applicable | Page 7 | Leprosy cases registered in the Notifiable Disease Information System (SINAN) from 2008 to 2018 of residents of the city of Cuiabá were included. SINAN is the Brazilian information system responsible for recording and processing information on mandatory notifiable diseases such as leprosy throughout Brazil, providing bulletins and reports of morbidity and constituting one of the main surveillance systems in the country.  The selected variables were: date of notification of the case, gender (male, female), age, race/ skin color (white, black, yellow, mixed race and indigenous), education (no schooling, incomplete elementary education, complete elementary education, incomplete high school education, complete high school education, incomplete higher education and complete higher education), WHO operational classification (Paucibacillary, Multibacillary), clinical form based on Madrid classification (indeterminate, tuberculoid, borderline and lepromatous) and assessment of grade of disability in the diagnosis (Grade 0 disability [G0D], Grade 1 disability [G1D], Grade 2 disability [G2D] and not evaluated). A total of 9,739 leprosy cases were reported between 2008 and 2018. As shown in Table 1, the majority of cases were male (58.37%), with a predominant age of 15 to 59 years (87.55%). Regarding the race/skin color variable, the majority declared themselves mixed race (61.08%), and the predominant level of education was incomplete elementary school (43.96%). All the clinical and social epidemiological characteristics of the cases diagnosed with leprosy is shown in the Table 1. |
| Data sources/ measurement | 8* | For each variable of interest, give sources of data and details of methods of assessment (measurement). Describe comparability of assessment methods if there is more than one group | Page 7-9 | The variables of interest were standardized, with data relating to age, gender, race/skin color and education considered in the sociodemographic dimension and operational classification, clinical form and Grade of Disability at diagnosis in the clinical-epidemiological dimension. The descriptive analysis of the sociodemographic and clinical epidemiological variables was performed, with the calculation of absolute frequencies (*n*) and relative frequencies (%).The construction of the time series, according to detection in general, by gender, age and Grade of Disability, the evolutions of the trends were calculated using the Seasonal-Trend decomposition procedure based on Loess (STL) [26]. This methodology is based on the classic decomposition of time series that disaggregates the total series into three additive components (trend, seasonality and error), allowing each of these components to be separately estimated and identifying the source of variability of the series in a more concise way than through a global analysis of the series. The STL procedure employs a succession of Loess smoothers, which consist of polynomial regressions locally weighted at each point in the series, in which the explanatory variables are the values closest to the response value to be estimated [27].  Having estimated the three components of the time series, only the trend was selected to characterize the behavior of the variables of interest over time. Subsequently, the Average Monthly Percentage Change (AMPC) was calculated for the trends in the general detection rates, by gender and cases with Grade of Disability, identifying the mean percentages and how much the trends increased or decreased over the study period. Finally, the trend in the rate of detection of Grade of Disability at the leprosy diagnosis and the trend of each Grade of Disability, both by age group, were also analyzed. |
| Bias | 9 | Describe any efforts to address potential sources of bias | Page 7-9 | Initially, the variables of interest were standardized, with data relating to age, gender, race/skin color and education considered in the sociodemographic dimension and operational classification, clinical form and Grade of Disability at diagnosis in the clinical-epidemiological dimension. The descriptive analysis of the sociodemographic and clinical epidemiological variables was performed, with the calculation of absolute frequencies (*n*) and relative frequencies (%).  Next, the time series of leprosy cases were constructed according to the total number of cases, gender (male and female) and Grade of Disability (G0D, G1D, G2D and not evaluated). Time series correspond to the set of observations ordered in time and recorded at regular periods, the aim of which is to identify patterns (increase, decrease, stationary) in a variable of interest over time [24,25]. For the construction of the time series, the general rates of detection and those stratified by gender were calculated, considering the total population of the municipality (for general detection rate) and the populations of men and women (for rates stratified according to gender) as the denominator, all with a multiplication factor per 100,000 inhabitants. For cases with Grade of Disability the rates were not calculated as it was decided to consider the total number of cases in the construction of their time series.  After the construction of the time series, according to detection in general, by gender, age and Grade of Disability, the evolutions of the trends were calculated using the Seasonal-Trend decomposition procedure based on Loess (STL) [26]. This methodology is based on the classic decomposition of time series that disaggregates the total series into three additive components (trend, seasonality and error), allowing each of these components to be separately estimated and identifying the source of variability of the series in a more concise way than through a global analysis of the series. The STL procedure employs a succession of Loess smoothers, which consist of polynomial regressions locally weighted at each point in the series, in which the explanatory variables are the values closest to the response value to be estimated [27].  One of the advantages of this methodology is that it is quite robust regarding the existence of outliers (present in the series, Figs.2-5). Trend refers to the general direction in which the variables of the time series develop, according to a time interval, presenting a pattern of increase/decrease of the variable over a certain period. Seasonality is identical patterns that a time series seems to obey and that occur regularly at fixed periods of time. Finally, noise is the fluctuations that occur over the time of the series, being irregular and random movements perceptible only with the removal of the other components [28].  Having estimated the three components of the time series, only the trend was selected to characterize the behavior of the variables of interest over time. Subsequently, the Average Monthly Percentage Change (AMPC) was calculated for the trends in the general detection rates, by gender and cases with Grade of Disability, identifying the mean percentages and how much the trends increased or decreased over the study period. Finally, the trend in the rate of detection of Grade of Disability at the leprosy diagnosis and the trend of each Grade of Disability, both by age group, were also analyzed. All analyses were performed using the R Studio® version 3.5.2 statistical software. |
| Study size | 10 | Explain how the study size was arrived at | Page 6 | All new leprosy cases reported to SINAN (Notifiable Diseases Information System) between 2010 and 2018 were selected. |

Continued on next page

| Quantitative variables | 11 | Explain how quantitative variables were handled in the analyses. If applicable, describe which groupings were chosen and why | Page 7- 9 | Initially, the variables of interest were standardized, with data relating to age, gender, race/skin color and education considered in the sociodemographic dimension and operational classification, clinical form and Grade of Disability at diagnosis in the clinical-epidemiological dimension. The descriptive analysis of the sociodemographic and clinical epidemiological variables was performed, with the calculation of absolute frequencies (*n*) and relative frequencies (%).  Next, the time series of leprosy cases were constructed according to the total number of cases, gender (male and female) and Grade of Disability (G0D, G1D, G2D and not evaluated). Time series correspond to the set of observations ordered in time and recorded at regular periods, the aim of which is to identify patterns (increase, decrease, stationary) in a variable of interest over time [24,25]. For the construction of the time series, the general rates of detection and those stratified by gender were calculated, considering the total population of the municipality (for general detection rate) and the populations of men and women (for rates stratified according to gender) as the denominator, all with a multiplication factor per 100,000 inhabitants. For cases with Grade of Disability the rates were not calculated as it was decided to consider the total number of cases in the construction of their time series.  After the construction of the time series, according to detection in general, by gender, age and Grade of Disability, the evolutions of the trends were calculated using the Seasonal-Trend decomposition procedure based on Loess (STL) [26]. This methodology is based on the classic decomposition of time series that disaggregates the total series into three additive components (trend, seasonality and error), allowing each of these components to be separately estimated and identifying the source of variability of the series in a more concise way than through a global analysis of the series. The STL procedure employs a succession of Loess smoothers, which consist of polynomial regressions locally weighted at each point in the series, in which the explanatory variables are the values closest to the response value to be estimated [27].  One of the advantages of this methodology is that it is quite robust regarding the existence of outliers (present in the series, Figs.2-5). Trend refers to the general direction in which the variables of the time series develop, according to a time interval, presenting a pattern of increase/decrease of the variable over a certain period. Seasonality is identical patterns that a time series seems to obey and that occur regularly at fixed periods of time. Finally, noise is the fluctuations that occur over the time of the series, being irregular and random movements perceptible only with the removal of the other components [28].  Having estimated the three components of the time series, only the trend was selected to characterize the behavior of the variables of interest over time. Subsequently, the Average Monthly Percentage Change (AMPC) was calculated for the trends in the general detection rates, by gender and cases with Grade of Disability, identifying the mean percentages and how much the trends increased or decreased over the study period. Finally, the trend in the rate of detection of Grade of Disability at the leprosy diagnosis and the trend of each Grade of Disability, both by age group, were also analyzed. All analyses were performed using the R Studio® version 3.5.2 statistical software. |
| --- | --- | --- | --- | --- |
| Statistical methods | 12 | (*a*) Describe all statistical methods, including those used to control for confounding | Not applicable | It is an ecological study |
|  |  | (*b*) Describe any methods used to examine subgroups and interactions | Not applicable | It is an ecological study |
|  |  | (*c*) Explain how missing data were addressed | Not applicable | It is an ecological study |
|  |  | (*d*) *Cohort study*—If applicable, explain how loss to follow-up was addressed  *Case-control study*—If applicable, explain how matching of cases and controls was addressed  *Cross-sectional study*—If applicable, describe analytical methods taking account of sampling strategy | Not applicable | Not applicable |
|  |  | (*e*) Describe any sensitivity analyses | Not applicable | Not applicable |
| Results | | | | |
| Participants | 13* | (a) Report numbers of individuals at each stage of study—eg numbers potentially eligible, examined for eligibility, confirmed eligible, included in the study, completing follow-up, and analysed | Not applicable | Not applicable |
|  |  | (b) Give reasons for non-participation at each stage | Not applicable | Not applicable |
|  |  | (c) Consider use of a flow diagram | Not applicable | Not applicable |
| Descriptive data | 14* | (a) Give characteristics of study participants (eg demographic, clinical, social) and information on exposures and potential confounders | Pages 9-10 | A total of 9,739 leprosy cases were reported between 2008 and 2018. As shown in Table 1, the majority of cases were male (58.37%), with a predominant age of 15 to 59 years (87.55%). Regarding the race/skin color variable, the majority declared themselves mixed race (61.08%), and the predominant level of education was incomplete elementary school (43.96%). Table 1 shows the clinical and social epidemiological characteristics of the cases diagnosed with leprosy in the scenario. |
|  |  | (b) Indicate number of participants with missing data for each variable of interest | Not applicable | There were no participants with missing data for each variable of interest. |
|  |  | (c) *Cohort study*—Summarise follow-up time (eg, average and total amount) | Not applicable | Not applicable |
| Outcome data | 15* | *Cohort study*—Report numbers of outcome events or summary measures over time | Not applicable | Not applicable |
|  |  | *Case-control study—*Report numbers in each exposure category, or summary measures of exposure | Not applicable | Not applicable |
|  |  | *Cross-sectional study—*Report numbers of outcome events or summary measures | Not applicable | Not applicable |
| Main results | 16 | (*a*) Give unadjusted estimates and, if applicable, confounder-adjusted estimates and their precision (eg, 95% confidence interval). Make clear which confounders were adjusted for and why they were included | Not applicable | Not applicable |
|  |  | (*b*) Report category boundaries when continuous variables were categorized | Not applicable | Not applicable |
|  |  | (*c*) If relevant, consider translating estimates of relative risk into absolute risk for a meaningful time period | Not applicable | Not applicable, it would be appropriated in other study design like cohort studies |

Continued on next page

| Other analyses | 17 | Report other analyses done—eg analyses of subgroups and interactions, and sensitivity analyses | Not applicable | Not applicable to this study. |
| --- | --- | --- | --- | --- |
| Discussion | | | | |
| Key results | 18 | Summarise key results with reference to study objectives | Page 13 | The present study aimed to investigate the epidemiological situation of leprosy, its behavior over the years and whether there were trends, both in the detection of the disease and in disabilities, analyzing disparities and or differences regarding gender and age in a hyperendemic metropolis in the Central-West region of Brazil. The study showed that leprosy has been declining in the scenario under study, however, when analyzed according to gender, we observed that the detection was increasing in men, however decreasing in women, which shows a gender inequity. The detection rate was also found to be increasing in the adult and economically active population, however, it was decreasing in children and adolescents (under 15 years of age) and in older adults (aged 60 years or more). Considering the data regarding disabilities, it was found that grade 0 disabilities were decreasing, however, grades 1 and 2 were increasing, which raises the hypothesis of late diagnosis and or underreporting. |
| Limitations | 19 | Discuss limitations of the study, taking into account sources of potential bias or imprecision. Discuss both direction and magnitude of any potential bias | Page 20 | The limitation of this study is the fact that the database used is secondary, so it may contain the presence of inconsistent information regarding quantity and quality with presence of data that was potentially ignored or incomplete. |
| Interpretation | 20 | Give a cautious overall interpretation of results considering objectives, limitations, multiplicity of analyses, results from similar studies, and other relevant evidence | Page 19-20 | This is the first study that sought to comprehend the situation of leprosy according to gender and age, showing that the disease does not behave in a homogeneous way among groups, but painfully affects the more vulnerable people. The findings show that the disease is growing in men, with this gender being most affected by more severe disabilities, which denotes their difficulty in accessing health services. The detection of leprosy in women has also been decreasing, however, the number of disabilities, although on a smaller scale than in men, has been increasing, indicating that this requires different approaches for the genders, in both promotion actions and the diagnosis.  The findings also highlighted another issue of concern, the growing number of disabilities in children and older adults, and these segments should be priorities for policies and social protection actions by the State, given that, in these stages of life they are in situations of vulnerability, therefore requiring State protected. In general, the studies carried out on leprosy, including the WHO reports, sought to generally analyze the behavior of the disease in the general population, always showing a decrease in the disease as a result, which is true, and which was confirmed in the present study. However, when stratifying by vulnerable segments, we still observe great inequalities and disparities in the context of leprosy. Accordingly, if there is a clear intention to overcome leprosy, investments in health and different methodological approaches must be considered, in both research and in the surveillance system of health systems and territories. The elimination of leprosy involves comprehending the differences in gender issues and life cycles in the health territories, and the elaboration of intervention projects aimed at overcoming the problem must be sufficiently supported by evidence from these approaches. Guiding public policies based on this evidence is essential for advancing equity and eliminating leprosy. |
| Generalisability | 21 | Discuss the generalisability (external validity) of the study results | Page 14-16 | The literature shows that the appearance of physical disabilities in people affected by leprosy are indications of late diagnosis [29], which may be due to the lack of knowledge of the population about the signs and symptoms suggestive of the disease, ignorance on the part of healthcare providers, difficulties related to early diagnosis, and/or the silent evolution of the disease. It should also be mentioned that stigma and discrimination continue to hinder spontaneously seeking diagnosis and treatment for the disease [30]. These disabilities can lead to permanent sequelae, as they result from injuries to the peripheral nerves responsible for the sensation of pain, touch and heat, in addition to the possible impairment of the vision of the person affected, making the individual susceptible to various accidents and injuries [31].  The result that men are more affected than women has also been found in other published works [32,33]. This can be related to several factors, such as greater exposure to risky situations, less concern about their own health and difficulties for men to access public health services [34,35]. The male population’s lack of self-care has been recorded in the literature since the 1990s, and currently there are few health policies aimed at this population in order to meet their needs. Barriers related to the difficulty of access to health services, the incompatibility between the hours of operation of health units and the workday and the belief of less susceptibility in relation to women contribute to this greater burden of leprosy in the male population [36].  Most of the cases had incomplete elementary education, an indicator of low schooling, which may be related to the social aspect and living conditions. Low levels of education hinder access to better jobs and better economic conditions [37,38]. Regarding race/skin color, the highest percentage of new cases was in people of mixed race, followed by white people. The variable race or ethnicity is still little considered in the context of leprosy and in studies where it has been evaluated, it was related more to the socioeconomic characteristics of the study region than to the disease itself [25,32]. Biologically, there is no evidence that race or ethnicity is an individual determinant of risk for the development of leprosy, however, in Brazil these characteristics are more related to the social inequalities of the context in which these populations are inserted. The mixed race and black populations, historically subjected to higher levels of social inequality, are more vulnerable to being affected by leprosy [2,25,32,39].  In this study, most of the cases were multibacillary, with the borderline clinical form. A higher proportion of multibacillary cases among new leprosy cases has been widely reported in endemic disease settings [40-42]. The predominance of multibacillary cases, with the most severe clinical forms (especially borderline and lepromatous cases), may suggest the occurrence of active transmission of the disease, and consequently, a greater potential to incapacitate the affected individuals [43].  Considering the time series in relation to the global detection rate, there was a clear downward trend from 2008 to mid-2012. After this time there was a clear increase until 2015, followed by a clear decrease until 2016. It then started increasing in 2017 and stabilized in 2018. From the year 2012 until the year 2016, the behavior with rapidly increasing and decreasing peaks was observed in the global detection rate, relatively decreasing in relation to the rest of the years studied. This behavior was also reflected in all the other series that followed, with the exception of the series with the population under 15 years of age. Specifically regarding detection in children under 15, despite ending the period with a decreasing trend, in 2015 there was a marked increase in detection.  In the state of Mato Grosso, in 2015, the “National Campaign for Leprosy, Geohelminthiasis and Trachoma” was initiated, which mobilized local health services to carry out actions related to the active search for cases, focusing on schoolchildren, aged from 5 to 14 years. The campaign was carried out in approximately 915 schools in 65 municipalities (including Cuiabá), to examine and treat more than 291.2 thousand students and possibly their contacts [22]. It is estimated that the campaign may have had an impact in the region studied, reflecting the peak of detection verified in the study for this age group.  Also in relation to children under 15, when analyzing the Grade of Disability, there were growing trends in the number of G1D and G2D in this age group, which may indicate that the municipality faces difficulties in the early diagnosis of the disease. A study by Xavier et al. (2014) [44] with children under 15 years of age indicated that the early exposure to the pathogen potentiates the chance of developing disabilities, showing that physical disabilities in this age group indicate a late diagnosis and prolonged exposure. People who are affected by multibacillary forms of the disease also have a greater chance of developing health problems [45]. Leprosy is highly disabling when not properly treated in this population, which can influence academic performance and cause problems related to social limitations, self-esteem and stigma experienced by the affected person, especially because this is a period of growth and physical and emotional development [46,47]. This age group is indicative of foci of transmission of recent infection, due to the characteristic of the disease being transmitted by lengthy exposure to the bacillus without adequate treatment [48]. |
| Other information | |  | | |
| Funding | 22 | Give the source of funding and the role of the funders for the present study and, if applicable, for the original study on which the present article is based | Page 20 | The authors would like to thank the Health Surveillance Service of the Cuiabá Regional Health Management Unit, of the state government of Mato Grosso for making the data available. |

*Give information separately for cases and controls in case-control studies and, if applicable, for exposed and unexposed groups in cohort and cross-sectional studies.

**Note:** An Explanation and Elaboration article discusses each checklist item and gives methodological background and published examples of transparent reporting. The STROBE checklist is best used in conjunction with this article (freely available on the Web sites of PLoS Medicine at http://www.plosmedicine.org/, Annals of Internal Medicine at http://www.annals.org/, and Epidemiology at http://www.epidem.com/). Information on the STROBE Initiative is available at www.strobe-statement.org.
