# Supplementary figures and images for: Inequality of gender, age and disabilities due to leprosy and trends in a hyperendemic metropolis: Evidence from an eleven-year time series study in Central-West Brazil
Source: PLoS Negl Trop Dis. 2021 Nov 16;15(11):e0009941. doi: 10.1371/journal.pntd.0009941 (PMC8631739; doi:10.1371/journal.pntd.0009941)

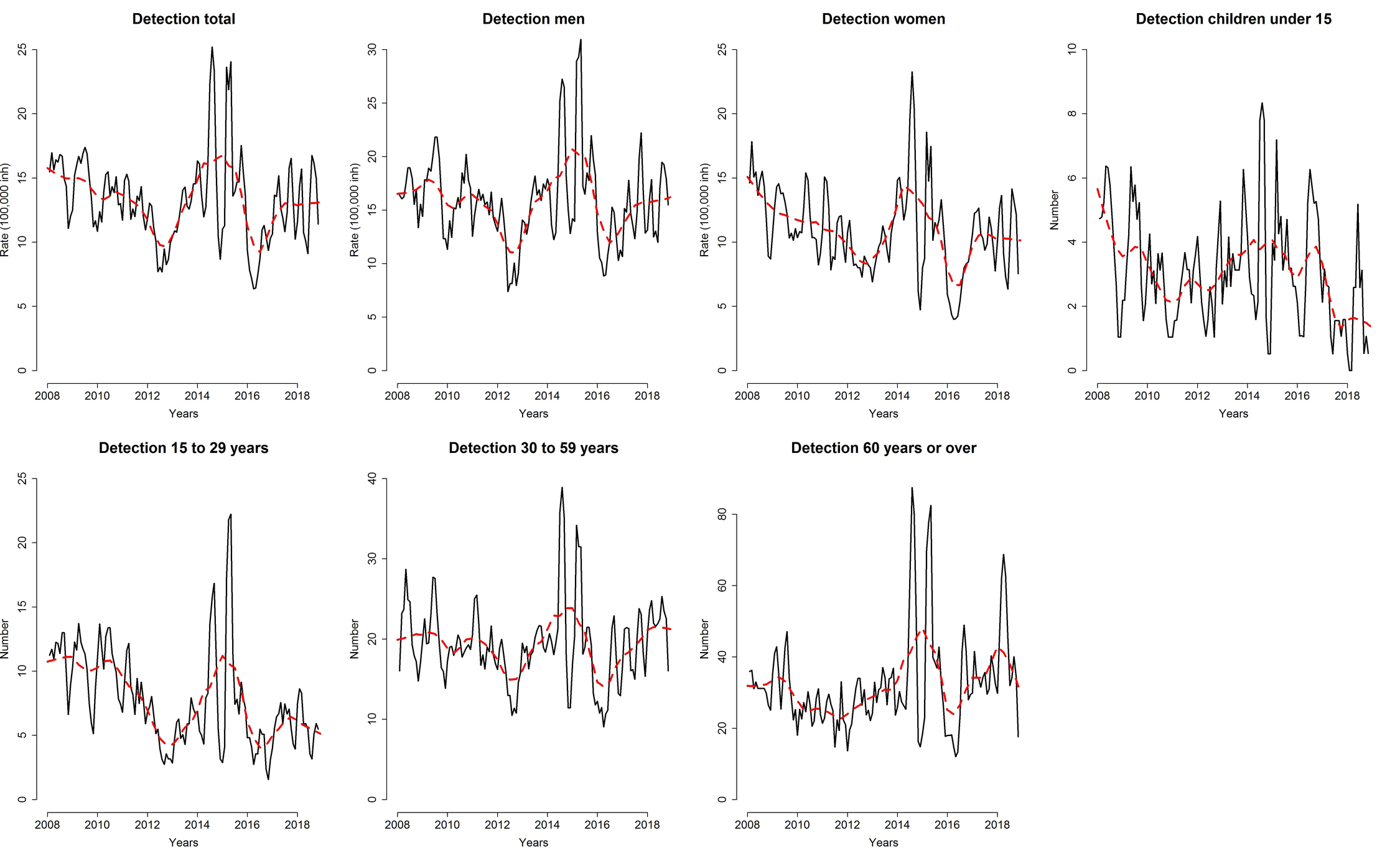

Supplement: S1 Fig — (Black line) Time series; (Red line) Trend. (TIF) [file pntd.0009941.s003.tif]

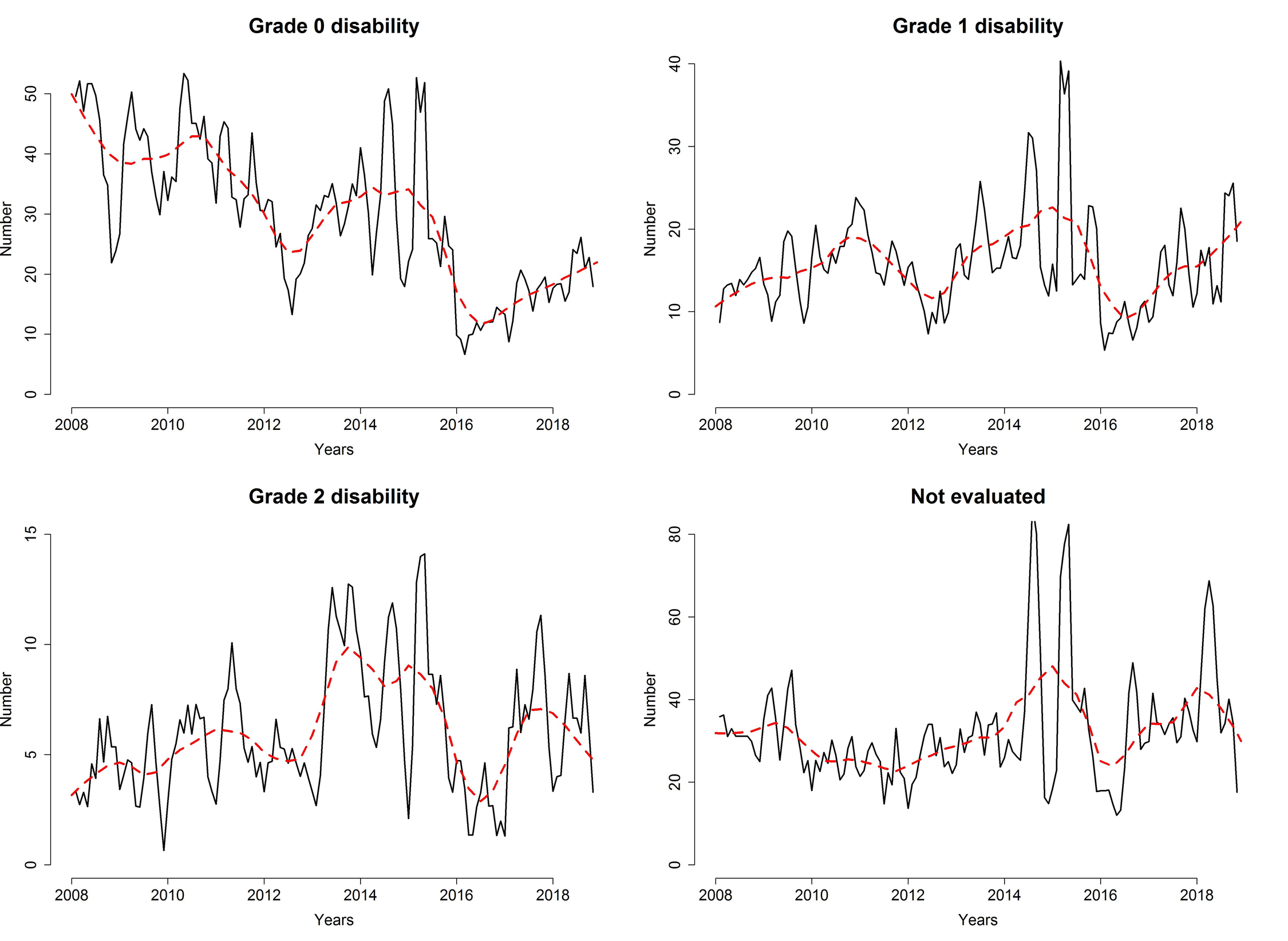

Supplement: S2 Fig — (Black line) Time series; (Red line) Trend. (TIF) [file pntd.0009941.s004.tif]

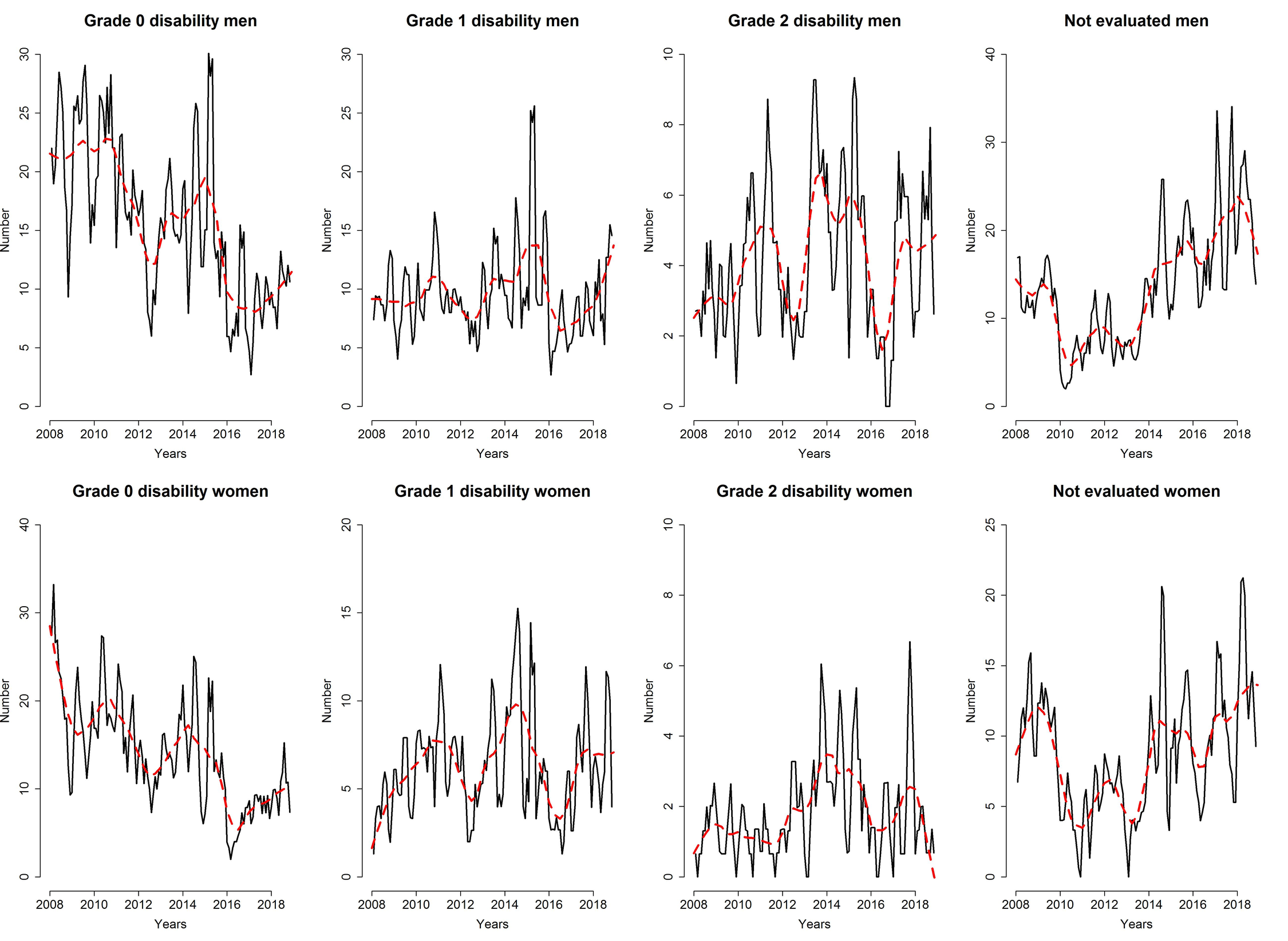

Supplement: S3 Fig — (Black line) Time series; (Red line) Trend. (TIF) [file pntd.0009941.s005.tif]

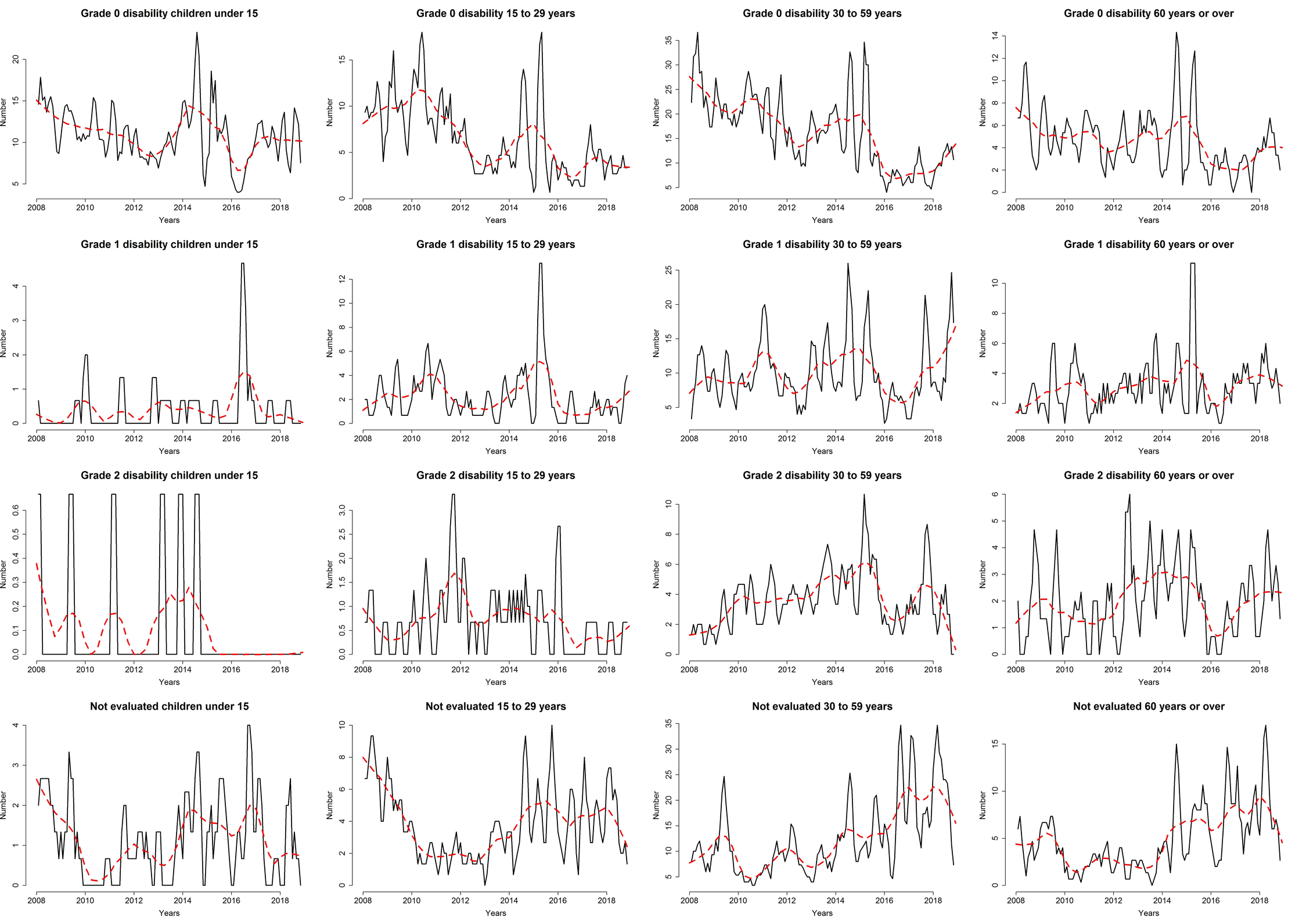

Supplement: S4 Fig — (Black line) Time series; (Red line) Trend. (TIF) [file pntd.0009941.s006.tif]
