# Supplementary material for: Inequality of gender, age and disabilities due to leprosy and trends in a hyperendemic metropolis: Evidence from an eleven-year time series study in Central-West Brazil
Source: PLoS Negl Trop Dis. 2021 Nov 16;15(11):e0009941. doi: 10.1371/journal.pntd.0009941 (PMC8631739; doi:10.1371/journal.pntd.0009941)
Supplement: Attachment — Submitted filename: Response to Reviewers.docx [file pntd.0009941.s007.docx]

Dear Editor,

Many thanks for your reply and your reviewers' comments about our manuscript “PNTD-D-21-00361Inequality of gender, age and disabilities due to leprosy and trend in a hyperendemic metropolis: Evidence from an eleven-year time series study in Central-West Brazil”.

The comments were appropriate to qualify/ improve the manuscript. We have forwarded a letter with the changes made in the manuscript, presenting our response for each comment from reviewers. Many thanks for your comments.

In relation to Fig 1, it was constructed using data available from a Brazilian public repository, the Brazilian Institute of Geography and Statistics (IBGE) (<https://www.ibge.gov.br/en/statistics/downloads-statistics.html>), open access, without restrictions and without copyright. The data used for making the map (Fig 1) refer to files in the Shapefile extension, which were manipulated through the QGis Desktop 3.14.0 software for making the map, with the authors being entirely responsible for drawing the figure.

In the lines 57-71 we add the Author summary between the Abstract and the Introduction, with 181 words long.

We’ve sent the manuscript to a professional academic proofreading service to review the English language as suggested by the authors.

The authors tried their best to reduce the discussion but at the same time trying to respond to the reviewers who requested the inclusion of more information.

Below, we respond point by point to the comments of all Reviewers.

Kind regards,

Martoreli Júnior et al.

**Reviewers comments:**

**Reviewer #2:**

**ResultsThe result section is clearly written. I would suggest to use not only colours, but also a different line appearance in the table to make them more clear for readers who are printing the tables in black/white. Please also see my comments below on the Results section.**

**Authors:** We’ve modified the tables to make them more clear for readers with different lines appearance.

**Conclusions**

**In the discussion, I am missing the role of leprosy prevention as post-exposure chemoprophylaxis with a single dose of rifampicin (SDR-PEP) for contacts of leprosy contacts, in combination with contact screening / active case finding. This is a very important strategy for decreasing the number of new cases / case detection delay / disability etc. For example in Brazil, a current research project is ongoing studying advanced chemoprophylaxis regimen (PEP++). But the evidence from e.g. COLEP & LPEP regarding SDR-PEP is clear and it is also included in the WHO Leprosy Guidelines for the Diagnosis, Treatment and Prevention of Leprosy. You could add this as recommendation**

**Authors:** The authors write a paragraph about SDRP and LPEP one lines 386 – 391:

“A current ally to fight leprosy is post-exposure leprosy prophylaxis (LPEP). According to the WHO in its guideline, the dose of rifampicin to single-dose rifampicin (SDR) can be used in children and adults [1]. In a double-blind study, SDR was found in leprosy contacts associated with a reduction in leprosy risk of 57% in 2 years and 30% in 5 -6 years [37]. As leprosy is a highly stigmatized disease, care should be taken when implementing this therapy in contacts, especially outside the patient's family.”

And add this as recommendations on lines 558 – 560:

“As was mentioned, LPEP and SDR could be studied by managers to implement them based on the WHO’s recommendations and their effectiveness as reported in the literature [1, 59].”

59.	Moet FJ, Pahan D, Oskam L, Richardus JH; COLEP Study Group. Effectiveness of single dose rifampicin in preventing leprosy in close contacts of patients with newly diagnosed leprosy: cluster randomized controlled trial. BMJ. 2008;336(7647):761–4. doi: 10.1136/bmj.39500.885752.BE.

.

**Including 1 study limitation may be a bit limited.**

**Authors:** Rewritten lines 561 – 570:

“A limitation of this study is that the database used is secondary, so it may contain inconsistent information regarding quantity and quality with presence of data that were potentially ignored or incomplete, data regarding the operational classification and Clinical form were, in some cases, not filled completely and or was filled in a dubious way. Another limitation involved STL, which is only a visual resource for showing findings and does not having a "measure" of increase or decrease. AMPC is based on a percentage, which is why does not contain a p-value or 95% confidence interval. The number of children with G2D in the trends is small and thus might not be able to show a clear trend. The interpretation of these data cannot be understood at the individuals’ level.

.”

**Abstract**

**32-35: First sentence in abstract is quite long, change this into 2 sentences to increase readability.**

**Authors:** Changed for 2 sentences.

Modified line 32-36: “The present study aimed to investigate the epidemiological situation of leprosy, in a hyperendemic metropolis in the Central-West region of Brazil. We studied it´s behavior over eleven years and whether there were trends, both in the detection of the disease and in disabilities, analyzing disparities and/or differences regarding gender and age.”

**35-36: Second sentence in abstract is incorrect. Start the sentence with e.g. “This is a …”**

**Authors:** Modified line 36: “This is a ecological time series study…’’

**45-46: In the abstract it is stated “Regarding detection according to gender, there was a decrease among women”, but “by evidencing a increasing trend of leprosy cases among women” (47-48). This seems contradictive, please make some changes. It should also be ”aN increasing trend”.**

**Authors:** Modified in line 45-49: “Regarding detection according to gender, there was a decrease among women and an increase in men. There was a trend towards an increase in disabilities in both genders. The study advances the knowledge by evidencing an increasing trend in disabilities in both genders, which may be related to the delay in diagnosis.”

**Introduction**

**60: In introduction, I would advise to change “which lead to physical disabilities” into “which can lead to physical disabilities”.**

**Authors**: Changed by “which can lead to physical disabilities…” in the line 61.

**61: Please remove “skin contact” in “skin contact or other means cannot be excluded”, the evidence for skin contact is low (i.e. using a tattoo needle in a leprosy patch and next tattooing another person with the same needle is risky, but not normal skin contact and rare of course) and stating it like this can sound stigmatizing for some people.**

**Authors**: In the line 62: deleted “skin contact or…”.

**65: Reference numbers should be placed before/after (depending on reference style) punctuation marks. In this case: before a comma (,) or full stop/period (.), not in the middle of a sentence. Please move [2,3] to after “imagined”.**

**Authors**: Moved [2,3] to after “imagined” in the line 64.

“…distant than imagined [2,3].”

**67: “In 2016, according to WGHO data, 143 countries…” -> This data is a bit outdates, especially because your data reflected in the time series study is ranging from 2008-2018. Please use WHO data from 2018 (or even 2019) here.**

**Authors:** We actualized the data with WHO 2019

Lines 65-69 “According to WHO data in the 2019 report, Brazil, India and Indonesia reported >10,000 new cases, classifying them as the three most highly endemic countries [4]. In 2019 Brazil had a detection rate of 13,23 per 100,000 inhabitants [5].”

4.	WHO 2020 Weekly epidemiological record Global leprosy (Hansen disease) update, 2019: time to step-up prevention initiatives 4 SEPTEMBER 2020, 95th YEAR / 4 SEPTEMBRE 2020, 95e ANNÉE No 36, 2020, 95, 417–440 Available from: https://www.who.int/publications/i/item/who-wer9536

5.	Ministério da Saúde Secretaria de Vigilância em Saúde Hanseníase 2021 Available from: <https://www.gov.br/saude/pt-br/media/pdf/2021/fevereiro/12/boletim-hanseniase-_-25-01.pdf>

**72: It seems like you are referring to both North and South America, so it should be continentS.**

**Authors:** Rewritten.

**77: “preventing cases with grade of disability” should be “preventing cases with disabilities”.**

**Authors:** Modified line 74: ‘’…preventing cases with disabilities.’’

**79: Please remove the capital at “The Plan” -> “The plan” or even better, change it to “The strategy”.**

**Authors:** Modified line 84: deleted plan and add the strategy

**89: Place a comma after “2010-2012”**

**Authors:** Placed a comma after “2010-2012”.

**102-107: This is also the case because you cannot count ‘undetected cases’.**

**Authors: Included “**because you cannot count ‘undetected cases”.

Modified line 109: “…WHO recognizes the disability grade indicator as the most sensitive measure of the real leprosy situation in a community and because we cannot count “undetected cases”, expressing, therefore, the fragility of the services in detecting the disease early [1].”

**105: “disabilitie” should be “disability”.**

**Authors:** Modified.

**111: the word “public” is missing: “define public policies” -> “define public health policies”.**

**Authors:** Modified line 116: “define public health policies”

**113: the word “tools” is missing: “more sensitive is the time series” -> “more sensitive tools is the time series”.**

**Authors:** modified line 118: add ‘’tools’’’ after “more sensitive”

**Methods**

**129: when including such an exact number as 3,266,538 km2, I would suggest to remove the word “approximately”.**

**Authors:** Removed “approximately”.

**138: Explain shortly what Gini index is.**

Modified line 143 “It also has a Gini index of 0.59, an indicator that measures how equitably a resource is distributed in a population; the nearest to 0 is the population with lowest inequality, and closest to 1 is the most unequal [18].”

18.	Frank A. Farris (2010) The Gini Index and Measures of Inequality, The American Mathematical Monthly, 117:10, 851-864, DOI: [10.4169/000298910X523344](https://doi.org/10.4169/000298910X523344)

**140. full stop is missing after “[17,18]”
Authors:** Modified lines 147: add full stop.

**140-141: capital usage is incorrect, please check. I would  suggest to remove most of the capitals used in this sentence.**

**Authors:** Removed all capital usage in this sentence as suggested.

**145-146: same point regarding the capitals, I would suggest to remove most of the capitals used in this sentence.**

**Authors:** Removed the capitals used in the sentence in the lines 141-156.

**153-154: which expectations are meant here? Where does 75.00% come from?
Authors:** According to the Notifiable Disease Information System (SINAN) the minimum amount of evaluation of new cases at the time of diagnosis should be at least 75.00% therefore 56,24% is not what we should expect.

Modified at line 157 – 165 “The municipality presents a detection rate of 45.30 cases for every 100,000 inhabitants, classifying it as hyper endemic for leprosy according to the Notifiable Disease Information System (SINAN) 2018, which classifies hyper endemic cities with a detection greater than 40 cases for every 100,000 [25,26] . In children under 15, in 2018 the detection rate was 7.9/100,000 inhabitants, placing the metropolis in the very high category for children. Regarding the disability grade, health units evaluated a mean of 56.24% of new cases at the time of diagnosis, below expectations, which should be at least 75.00% according to SINAN [25, 26].”

25.	Brasil. Prefeitura de Cuiabá. Boletim Epidemiológico Hanseníase. Cuiabá: SES; 2019. Available from: <http://201.24.3.67:8080/portal/upload/arquivos/20190523165956000163.pdf> (acessed 10 March, 2021)

26.	MINISTÉRIO DA SAÚDE, Roteiro para uso do Sistema de Informação de Agravos de Notificação – Sinan NET para hanseníase 2018. Available from: <http://portalsinan.saude.gov.br/images/documentos/Agravos/Hanseniase/Manual_tabulacao_dos_indicadores_de_hanseniase.pdf>

**Study population and information sources**

**164: I do understand why you included this (race/skin color), and the explanation on this in the discussion is very clear and correct. But when people are reading this in the Methods section, it is a bit in your face. Registering this data should be avoided or happen very carefully from an ethical perspective, as it may sound like ethical profiling / offensive / discriminatory to some people. The colors as outlined here (“white, black, yellow, mixed, and indigenous”) is not ethical preferred. Starting with white is tricky (also now with Black Lives Matter), alphabetical order would be better. I would include information that people had to register their own race (instead of the health professionals / researchers registering it, because it is hard to judge whether someone is mixed or black for example). I would remove it completely from the article. If you want to leave it in, I would suggest to more clearly explain also in the Methods section that this is registered in the national leprosy system and that this is outdated or not found to be ethical anymore and why this data can still be important and change/remove this listing of skin tones.**

**Authors:** Removed completely from the article as suggested.

**181/189/192/212/214/215 is sometimes written with capitals and sometimes without, I would suggest to write it without capitals. I also prefer “disability grade” over “grade of disability”. : Grade of Disability**

**Authors:** Rewritten without capitals and changed for disability grade.

**202-206: I would use “” or ‘’ for the words you are defining: ‘Trend’, ‘Seasonality’, ‘noise’.**

**Authors:** Used “or”.

**Results:**

**229 & Table & 321: see comments above about “race/skin color”**

**Authors:** Removed completely from the article.

**235 & 262: Delete “Source: Authors”. If there is no other source included, it is clear it is from the authors.**

**Authors:** Removed Source: Authors.

**238/240/241-242/245/Table 2/260/262/264/268/268/271/274/354/372: See comment above: “Grade of Disability” -> “disability grade”.**

**Authors:** Changed for disability grade.

**243: full stop missing after “Fig”.**

**Authors:** Add a full stop after “Fig”.

**Table 2: “General Population”-> “General population”.**

**Authors:** In the Table 2 changed for “General population”.

**Fig. 2/3/4/5: It may be nice to use strips/dots for one of the lines in the graphs (e.g. the red one) besides a color difference, so people who print it black & white can still see the color difference.**

**Authors:** Used a different line to readers who print it black & white can still see the difference.

**279 & 339 & 434: I would suggest a different word here, as “behavior” is more suitable for humans/animals, go for e.g. “trend” here instead.**

**Authors:** Deleted behavior and add “trend” in lines 325/503/518.

**280 & 289: “and or” should be “and/or”.**

**Authors:** Add and/or.

**297: remove “the” before “: disability”.**

**Authors:** Deleted ‘’the’’ in line 354.

**299: Remove “The”  before “literature”.**

**Authors:** Modified Line 346: deleted ‘’The’’

**300: Maybe add  reference here, as “literature” sounds like plural.**

**Authors:** Modified line 346: add a new reference.

34.	Gómez, L., Rivera, A., Vidal, Y., Bilbao, J., Kasang, C., Parisi, S., Schwienhorst-Stich, E.-M. and Puchner, K.P: Factors associated with the delay of diagnosis of leprosy in north-eastern Colombia: a quantitative analysis. Trop Med Int Health; 2018; 23(2): 193-198. Available from: <https://doi.org/10.1111/tmi.13023>

**308: Possibly remove “various”.**

**Authors:** Modified line 360: deleted ‘’various’’.

**310: you could replace “published works” by simply staying “studies”.**

**Authors:** Modified line 369: deleted published works, add ‘’studies’’

**311: what is meant here by “risky situations”? It this referring to getting infected? As prolonged contact is needed, I would not call this “risky situations” (also sounds a bit stigmatizing), or is it referring to possible damage to the hands because of work related accidents or so? I would delete “risky situations” or rephrase and explain it.**

**Authors:** Modified line 370: deleted ‘’risky situations’’

**313-314: at least 2 references seem to be missing, as you are talking about evidence from the 90ies and current policies.**

**Authors:** Modified line 371: add news 2 references in “The male population’s lack of self-care has been recorded in the literature since the 1990s [42].Currently there are few health policies aimed at this population in order to meet their needs [43].”

42.	Oliveira MHP, Romanelli G. Os efeitos da hanseníase em homens e mulheres: um estudo de gênero. Cad. Saúde Pública  [Internet]. 1998;  14( 1 ): 51-60. <https://doi.org/10.1590/S0102-311X1998000100013>.

43.	Couto MT, G R. Men, health and public policies: gender equality in question. Ciênc. saúde coletiva  [Internet]. 2012;  17( 10 ): 2569-2578.   <http://dx.doi.org/10.1590/S1413-81232012001000002>.

**343: add the word “the” between “regarding” and “detection”.**

**Authors:** Rewritten.

**343 & 354: add “years of age” after “15”.**

**Authors:** Modified line 416 and 432: add years of age

**348-349: word repetition “carry out”, if you like, change for executed/organized…**

**Authors:** Modified line 408: deleted carry out add executed

**357 & 377: reference location not correct (should be at end of sentence or before comma).**

**Authors:** Modified.

**357-360: what do you mean here by the statement that “early exposure” leads to disabilities?  Because of the length of the exposure/infection period (although, they were still children at time of diagnosis, so it cannot be decades for example) or is it because their bodies were still developing biologically at that age. Or both?**

**Authors:** Modified lines 419: “ A study by Xavier et al. (2014) with children under 15 years of age indicated that the early exposure to the pathogen in this age group suggests a late diagnosis and prolonged exposure [48]. People who are affected by multibacillary forms of the disease also have a greater chance of developing health problems and the fact that there are any G2D in children is concerning, as it falls far short of the WHO goal for zero disability in children [1, 48, 49].”

**362: I would change “academic performance” into “school performance”, as “academic” often refers to university level.**

**Authors:** Modified line 426: academic performance is deleted, school performance has added.

**383: delete commas around “as well as”.**

**Authors:** In line 452 deleted commas around as well as.

**384: “incapacity” –> “incapacities”.**

**Authors:** Modified to incapacities.

**400: change the word “scenario” into “setting”.**

**Authors:** Changed for setting.

**400: you may like to add “registration at health care level/national surveillance system”.**

**Authors:** Modified the sentence and add “registration at health care level/national surveillance system”

**402: you may like to add “lack of adherence”.**

**Authors:** Add lack of adherence

**408: “a way of” sounds a bit informal, you could replace it for “a method of”.**

**Authors:** Deleted ‘’ a way of’’ substuido por ‘’a method of’’

**421-422: change “does not behave in a homogenous way” into “disease trend is not homogenous”.**

**Authors:** Modified and deleted ‘’does not behave in a homogeneous way’’ for “trend is not homogeneous”.

**427: what is meant by “promotion actions”? Awareness raising? Self care? Please explain.**

**Authors:** Modified line 507: “The detection of leprosy in women has also been decreasing; however, the number of disabilities, although on a smaller scale than in men, has been increasing, indicating the need for different approaches for the genders, in both promotion actions and the diagnosis as awareness raising by the city’s health service and Primary Health Units. Also, this decreasing trend in women be due to insufficient recognition or stigma [37].”

37.	Price VG. Factors preventing early case detection for women affected by leprosy: a review of the literature. Glob Health Action. 2017 Jan-Dec;10(sup2):1360550. doi: 10.1080/16549716.2017.1360550.PMID: 28853325

**431 & 432: I don’t think “State” should be capitalized here.**

**Authors:** Removed capital from “state”.

**432: “State protection” has a double definition (also a negative one), I would replace it for “state support”**

**Authors:** Modified line 516: replaced state protection by state support.

.
**434: 432-435: references are missing, as you are referring to multiple studies and WHO reports.**

**Authors:** Rewritten lines 517-522 and add the references 1,4 and 15.

1 WHO 2018. Guidelines for the Diagnosis, Treatment and Prevention of Leprosy. Geneva: World Health Organization; 2018. Licence: CC BY-NC-SA 3.0 IGO. <https://apps.who.int/iris/handle/10665/274127> (acessed 10 March, 2021).

4 WHO 2020 Weekly epidemiological record Global leprosy (Hansen disease) update, 2019: time to step-up prevention initiatives 4 SEPTEMBER 2020, 95th YEAR / 4 SEPTEMBRE 2020, 95e ANNÉE No 36, 2020, 95, 417–440 https://www.who.int/publications/i/item/who-wer9536

15 Ramos ACV, Gomes D, Neto MS, Berra TZ, Assis IS, Yamamura M, et al. Trends and forecasts of leprosy for a hyperendemic city from Brazil’s northeast: Evidence from an eleven-year time-series analysis. PLoS One. 2020; 15(8):e0237165. <http://dx.doi.org/10.1371/journal.pone.0237165> PMID: 32764785

**435: delete “which is true”, as you need to have verified all these studies/data to know for sure.**

**Authors:** Deleted “which is true”.

**435: a decrease in new cases can also be caused by a lack of active case finding activities, and this, is not always a ‘positive’ finding.**

Authors: Rewritten the lines 520-522 “This result can also be caused by a lack of active case-finding activities, thus not always leading to a positive finding [1,4,15].”

**445: usually, multiple limitations (at least 2) are named**

**Authors:** Rewritten lines 537 – 546:

“A limitation of this study is that the database used is secondary, so it may contain inconsistent information regarding quantity and quality with presence of data that were potentially ignored or incomplete, data regarding the operational classification and Clinical form were, in some cases, not filled completely and**/**or was filled in a dubious way. Another limitation involved STL, which is only a visual resource for showing findings and does not having a "measure" of increase or decrease. AMPC is based on a percentage, which is why does not contain a p-value or 95% confidence interval. The number of children with G2D in the trends is small and thus might not be able to show a clear trend. The interpretation of these data cannot be understood at the individuals’ level.”

**Say something on post-exposure prophylaxis (PEP) as prevention strategy for leprosy (see my comment above) in the discussion section.**

**Authors:** The authors write a paragraph about SDRP and LPEP one lines 362 – 367:

“A current ally to fight leprosy is post-exposure leprosy prophylaxis (LPEP). According to the WHO in its guideline, the dose of rifampicin to single-dose rifampicin (SDR) can be used in children and adults [1]. In a double-blind study, SDR was found in leprosy contacts associated with a reduction in leprosy risk of 57% in 2 years and 30% in 5 -6 years [37]. As leprosy is a highly stigmatized disease, care should be taken when implementing this therapy in contacts, especially outside the patient's family.”

37.	Price VG. Factors preventing early case detection for women affected by leprosy: a review of the literature. Glob Health Action. 2017 Jan-Dec;10(sup2):1360550. doi: 10.1080/16549716.2017.1360550.PMID: 28853325

And add this as recommendations on lines 534 – 536:

“As was mentioned, LPEP and SDR could be studied by managers to implement them based on the WHO’s recommendations and their effectiveness as reported in the literature [1, 59].

59.	Moet FJ, Pahan D, Oskam L, Richardus JH; COLEP Study Group. Effectiveness of single dose rifampicin in preventing leprosy in close contacts of patients with newly diagnosed leprosy: cluster randomized controlled trial. BMJ. 2008;336(7647):761–4. doi: 10.1136/bmj.39500.885752.BE.

**References:**

**Reference 1  (457) and reference 5 are the same, though 2 difference websites are used. Please replace reference 1 with number 5 (2018 WHO is the correct reference for the WHO Leprosy Guidelines).**

**Authors:** Replaced the reference 1 with number 5.

**Reviewer #3**

**The analysis presented matches the analysis plan. The results are clearly and completely presented. But: A standardized population need to be provided and calulated for the age and gender groups.**

**Authors:** A standardized population applies among distinct populations with different age patterns. In the study we observed only one city through a period of time without a comparison between cities or other group of individuals, thus preferring to work with the gross and segmented rate according to gender and age.

**Please include the diagnosis criterion of leprosy for better understanding.**

**Authors:** Included in line 174-178:

“According to the Brasil Practice Guide about Leprosy, the cases are diagnosed if the person presents a defined area with altered or completely lost of sensitivity with or without compromised nerve, or nerves with neural thickening, and/or positive bacilloscopy, the bacilloscopy is not the defining factor but always the clinical and epidemiological evaluation [27].”

27.	MINISTÉRIO DA SAÚDE, Guia prático sobre a Hanseníase – Secretaria de Vigilância em Saúde – Departamento de Vigilância e Doenças Transmissíveis - Brasília - DF 2017 Availble from: https://portalarquivos2.saude.gov.br/images/pdf/2017/novembro/22/Guia-Pratico-de-Hanseniase-WEB.pdf

**Please clarify the mobile population for analysis since it could be a confounding factor for the result.**

**Authors:** The data was used based on 2010 accountability of residents of the city. On the line 169 I clarified that we only gather information of residents of Cuiabá thus there was not confounding factor related to mobile population:

“…Leprosy cases registered in the Notifiable Disease Information System (SINAN) from 2008 to 2018 of only residents of the city of Cuiabá…”

**Is there any policy change for leprosy which could influence the detection of leprosy.**

**Authors:** Rewritten paragraph 406:

“In the state of Mato Grosso, in 2015, the “National Campaign for Leprosy, Geohelminthiasis and Trachoma” was initiated, which mobilized local health services to executed actions related to the active search for cases, focusing on schoolchildren, aged from 5 to 14 years. The campaign was carried out in approximately 915 schools in 65 municipalities (including Cuiabá), to examine and treat more than 291.2 thousand students and possibly their contacts [22]. It is estimated that the campaign may have had an impact in the region studied, reflecting the peak of detection verified in the study for this age group. No further policies were encountered on public archives to influence the detection of leprosy on the region in the time period.”

**The method of Seasonal-Trend decomposition procedure based on Loess (STL) can be described more clearly for better understanding by readers.**

**Authors:** Described in the lines 216-221:

“The STL has a simple design that consists of a sequence of applications of the Loess smoother and allows analysis of the properties of the procedure and quick calculations even for a very long time series and large amounts of trend and seasonal smoothing, a very small amount of trend smoothing, and seasonal components that are not distorted by aberrant behavior in the data ability to decompose time series with missing values. [32].”

32. Theodosiou M. “Forecasting monthly and quarterly time series using STL decomposition”. Int J Forecast. 2011; 27(4):1178-1195. <https://doi.org/10.1016/j.ijforecast.2010.11.002>

**The authors need to discuss in more detail to help readers understanding the result.**

**Authors:** We re-elaborate the discussion to better understanding the results.

**Reviewer #4:**

**The first paragraph of the introduction is rather outdated. Please use 2019 WHO data and also refer to the latest WHO strategic document for leprosy.**

**Authors:** We used the following reference:

1. WHO 2018. Guidelines for the Diagnosis, Treatment and Prevention of Leprosy. Geneva: World Health Organization; 2018. Licence: CC BY-NC-SA 3.0 IGO. <https://apps.who.int/iris/handle/10665/274127>

**Please provide the definition used in Brazil for hyper endemic. For international use, consider to just state 'highly endemic' and use this term as well in the title, instead of 'hyper endemic'.
Authors:** Provided in the lines 157 – 160:

“The municipality presents a detection rate of 45.30 cases for every 100,000 inhabitants, classifying it as hyper endemic for leprosy according to the Notifiable Disease Information System (SINAN) 2018, which classifies hyper endemic cities with a detection greater than 40 cases for every 100,000 [25,26].”

25.	Brasil. Prefeitura de Cuiabá. Boletim Epidemiológico Hanseníase. Cuiabá: SES; 2019. Available from: <http://201.24.3.67:8080/portal/upload/arquivos/20190523165956000163.pdf> (acessed 10 March, 2021)

26.	MINISTÉRIO DA SAÚDE, Roteiro para uso do Sistema de Informação de Agravos de Notificação – Sinan NET para hanseníase 2018. Available from: <http://portalsinan.saude.gov.br/images/documentos/Agravos/Hanseniase/Manual_tabulacao_dos_indicadores_de_hanseniase.pdf>

**There is quite some 'fog' in relation to disability grade, its registration and the way it was dealt with in the analysis. Lines 152-154 are unclear; e.g. what does 'below expectations' mean. Also the sentence in lines 188-190 is unclear. What total number? And how does this translate in the results? There I see a breakdown by disability grade and 'not registered'. With the great uncertainty with regard to the indicator disability grade, why do you not just take 'grade 0' and 'joint grade 1 and 2' in the trend analysis, and ignore the 'not evaluated'?**

**Authors:** The author’s changed the sentence in the line 206-208:

“For the construction of time series of the cases with disability grade we considered all the number of cases with disability grade (G0D, G1D, G2D and not evaluated).”

The authors are grateful for the suggestion of the union between grades 1 and 2 but unfortunately we are not going to make it because these variables are used for the notification of leprosy in Brazil. And internationally the assessment of the disability grade (G0D, G1D, G2D and not evaluated) is performed and each degree of disability has its specificity and its clinical and epidemiological relevance regarding it’s uncertainty of filling in by third parties.

**I think very important information is missing necessary for interpreting trends, namely operational changes in the leprosy control program, or for that matter the health system in general. The total leprosy detection over the ten years is rather spiky. It comes down between 2008 and 2012, increases and decreases steeply between 2012 and 2016, and then increases again to reach possibly a plateau. The health system (or other operational changes) during this time need to be described in some detail in the methods section under a separate heading. This is essential context to understand and interprete the trends.**

**Authors:** We mentioned the structure of the city in methods in lines 147 -156:

“It also has 63 Primary Health Units, 4 Polyclinics, and 2 Emergency Care Units, distributed throughout 4 Administrative Regions north, south, east, and west, which have variable numbers of neighborhoods [21,22]. It should be highlighted that the municipality provides the following referral services for procedures of greater technological complexity as well as for leprosy, : CERMAC - (dermatology surveillance services), metropolitano hospital (hospital care for leprosy surgery services), Julio Muller university hospital (hospital care for ophthalmology referral), CRIDAC center specialized in rehabilitation (CER III), and regional outpatient and hospital referrals [23,24].”

23.	Brasil. Prefeitura Municipal de Cuiabá. Instituto de Planejamento e Desenvolvimento Urbano. Organização Geopolítica de Cuiabá; 2007.

And in the discussion we name an action that was found in the same period as the study was carried and rewritten in the paragraph 406:

“In the state of Mato Grosso, in 2015, the “National Campaign for Leprosy, Geohelminthiasis and Trachoma” was initiated, which mobilized local health services to executed actions related to the active search for cases, focusing on schoolchildren, aged from 5 to 14 years. The campaign was carried out in approximately 915 schools in 65 municipalities (including Cuiabá), to examine and treat more than 291.2 thousand students and possibly their contacts [22]. It is estimated that the campaign may have had an impact in the region studied, reflecting the peak of detection verified in the study for this age group. No further policies were encountered on public archives to influence the detection of leprosy on the region in the time period.”

24.	Brasil. Secretaria do Estado de Saúde - SES – MT. Assessoria da secretaria de saúde do estado do Mato Grosso. Campanha contra hanseníase, verminoses e tracoma termina dia 29. Brasília: SES-MT; 2015. <http://www.mt.gov.br/-/campanha-contra-hanseniase-verminoses-e-tracoma-termina-dia-29>

**To start with, cut the discussion by at least 50%. It is far too long and therefore unclear. In the discussion state first what you consider the really most important findings of your study. Which trends are really relevant in terms of being unexpected, undesired and amendable to health care interventions?**

**Authors:** The author’s tried to cut the discussion and make clearer focusing on the most important findings and discussing it based on the literature.

**After stating the most important findings, only then start with some explanation and interpretation. Make clear that you are discussing everything against the Brazilian background. You use the term 'global' at a certain point, but I understand it to mean Brazil as a whole and not the world. Here it is also important to take into account the operational factors, that you have now incorporated in the new figure 1.**

**Authors:** The authors stated some findings and discussed as in the lines 346-347:

“Literature shows that the appearance of physical disabilities in people affected by leprosy is an indications of late diagnosis [34]”

Discussed and stated more findings further more in the lines 348-361:

“The fact that the most serious cases occurred in children, young adults and older adults, demonstrates weaknesses of the services in the early detection of these cases and also the lack of knowledge of the population about the signs and symptoms suggestive of the disease, ignorance on the part of health care providers, difficulties related to early diagnosis, and/or the silent evolution of the disease. An important result was the significant reduction in cases with G0D, as well as an increasing trend in cases where disability was not evaluated at the time of diagnosis, revealing a weakness of the health services. These services should fight against the stigma and discrimination that continues to hinder the spontaneous seeking of a diagnosis and treatment for the disease [35]. These disabilities can lead to permanent sequelae, as they result from injuries to the peripheral nerves responsible for the sensation of pain, touch, and heat, in addition to the possible impairment of the vision of the person affected, making the individual susceptible to accidents and injuries [36].”

35.	Henry M, Galan N, Teasdale K, Prado R, Amar H, Rays MS, et al. Factors contributing to the delay in diagnosis and continued transmission of leprosy in Brazil - an explorative, quantitative, questionnaire based study. PLoS Negl Trop Dis. 2016; 10:e0004542. Available from: <https://doi.org/10.1371/journal.pntd.0004542> PMID: 26977811

36.	Seshadri D, Khaitan BK, Khanna N, Sagar R. Dehabilitation in the era of elimination and rehabilitation: a study of 100 leprosy patients from a tertiary care hospital in India. Lepr Ver. 2015; 86:62-74. Available from: <https://pubmed.ncbi.nlm.nih.gov/26065148/> PMID: 26065148

We’ve tried to state the findings and discuss about them as above in the entire discussion, and also make the discussion clear and take account operational factor with that in mind in the lines 458-467:

“The older adult age group (aged 60 years or more) had a decreasing trend in the rate of detection of new cases and increasing values in G1D and G2D. The antagonism between the decrease in the detection rate and the increase in disabilities is a strong indication of difficulties in the active search for cases and the possibility of underreporting [43,52].”

43.	Couto MT, G R. Men, health and public policies: gender equality in question. Ciênc. saúde coletiva  [Internet]. 2012;  17( 10 ): 2569-2578.   Available from: <http://dx.doi.org/10.1590/S1413-81232012001000002>.

52.	Croft RP, Nicholls PG, Steyerberg EW, Richardus JH, Cairns W, Smith S. A clinical prediction rule for nerve-function impairment in leprosy patients. Lancet. 2000; 355(9215):1603-1606. Available from: <https://doi.org/10.1016/s0140-6736(00)02216-9> PMID: 10821364

“The issue of not evaluating cases for disability showed an increasing trend over the study period. The assessment of physical disability at the time of diagnosis is a priority action for newly diagnosed leprosy cases, and the fact that “non-evaluation” presents an increasing trend may raise discussions regarding the management of leprosy cases in the research setting.”

And 531-536:

“The health system that determines strategic actions for leprosy in this region could focus on actions for the health care providers to correctly evaluate the disabilities. Active search actions can promote the new detection of cases, and the search and follow-up of their communicators must also be promoted. As was mentioned, LPEP and SDR could be studied by managers to implement them based on the WHO’s recommendations and their effectiveness as reported in the literature [1, 59].”

1.	WHO 2018. Guidelines for the Diagnosis, Treatment and Prevention of Leprosy. Geneva: World Health Organization; 2018. Licence: CC BY-NC-SA 3.0 IGO. Available from: <https://apps.who.int/iris/handle/10665/274127> (acessed 10 March, 2021).

59.	Moet FJ, Pahan D, Oskam L, Richardus JH; COLEP Study Group. Effectiveness of single dose rifampicin in preventing leprosy in close contacts of patients with newly diagnosed leprosy: cluster randomized controlled trial. BMJ. 2008;336(7647):761–4. doi: 10.1136/bmj.39500.885752.BE.

**Much of the disparities are due to delayed detection, which may apply more strongly to e.g. women and people in the working age. This is also related to stigma. Try to bring that together in the discussion more comprehensively, instead of just going along the different groups (gender, age and disability) in order of appearance in analysis.**

**Authors:** Authors rewritten the discussion and express this in the lines 346-357:

“Literature shows that the appearance of physical disabilities in people affected by leprosy is an indications of late diagnosis [34]. The fact that the most serious cases occurred in children, young adults and older adults, demonstrates weaknesses of the services in the early detection of these cases and also the lack of knowledge of the population about the signs and symptoms suggestive of the disease, ignorance on the part of health care providers, difficulties related to early diagnosis, and/or the silent evolution of the disease. An important result was the significant reduction in cases with G0D, as well as an increasing trend in cases where disability was not evaluated at the time of diagnosis, revealing a weakness of the health services. These services should fight against the stigma and discrimination that continues to hinder the spontaneous seeking of a diagnosis and treatment for the disease [35].”

34.	Gómez, L., Rivera, A., Vidal, Y., Bilbao, J., Kasang, C., Parisi, S., Schwienhorst-Stich, E.-M. and Puchner, K.P: Factors associated with the delay of diagnosis of leprosy in north-eastern Colombia: a quantitative analysis. Trop Med Int Health; 2018; 23(2): 193-198. Available from: <https://doi.org/10.1111/tmi.13023>

35.	Henry M, Galan N, Teasdale K, Prado R, Amar H, Rays MS, et al. Factors contributing to the delay in diagnosis and continued transmission of leprosy in Brazil - an explorative, quantitative, questionnaire based study. PLoS Negl Trop Dis. 2016; 10:e0004542. Available from: <https://doi.org/10.1371/journal.pntd.0004542> PMID: 26977811

**Whereas the general discussion text should be much shorter, the paragraph on strengths and weaknesses should be a more detailed.**

**Authors:** Rewritten on the paragraph 346:

“Literature shows that the appearance of physical disabilities in people affected by leprosy is an indications of late diagnosis [34]. The fact that the most serious cases occurred in children, young adults and older adults, demonstrates weaknesses of the services in the early detection of these cases and also the lack of knowledge of the population about the signs and symptoms suggestive of the disease, ignorance on the part of health care providers, difficulties related to early diagnosis, and/or the silent evolution of the disease. An important result was the significant reduction in cases with G0D, as well as an increasing trend in cases where disability was not evaluated at the time of diagnosis, revealing a weakness of the health services. These services should fight against the stigma and discrimination that continues to hinder the spontaneous seeking of a diagnosis and treatment for the disease [35]. These disabilities can lead to permanent sequelae, as they result from injuries to the peripheral nerves responsible for the sensation of pain, touch, and heat, in addition to the possible impairment of the vision of the person affected, making the individual susceptible to accidents and injuries [36].”

34.	Gómez, L., Rivera, A., Vidal, Y., Bilbao, J., Kasang, C., Parisi, S., Schwienhorst-Stich, E.-M. and Puchner, K.P: Factors associated with the delay of diagnosis of leprosy in north-eastern Colombia: a quantitative analysis. Trop Med Int Health; 2018; 23(2): 193-198. Available from: <https://doi.org/10.1111/tmi.13023>

35.	Henry M, Galan N, Teasdale K, Prado R, Amar H, Rays MS, et al. Factors contributing to the delay in diagnosis and continued transmission of leprosy in Brazil - an explorative, quantitative, questionnaire based study. PLoS Negl Trop Dis. 2016; 10:e0004542. Available from: <https://doi.org/10.1371/journal.pntd.0004542> PMID: 26977811

36.	Seshadri D, Khaitan BK, Khanna N, Sagar R. Dehabilitation in the era of elimination and rehabilitation: a study of 100 leprosy patients from a tertiary care hospital in India. Lepr Ver. 2015; 86:62-74. Available from: <https://pubmed.ncbi.nlm.nih.gov/26065148/> PMID: 26065148

**Finally, the conclusion is very general. Can you make it a bit more specific, based on your findings? Just one or two sentences more.**

**Authors:** The authors add lines 531 - 536.

“The health system that determines strategic actions for leprosy in this region could focus on actions for the health care providers to correctly evaluate the disabilities. Active search actions can promote the new detection of cases, and the search and follow-up of their communicators must also be promoted. As was mentioned, LPEP and SDR could be studied by managers to implement them based on the WHO’s recommendations and their effectiveness as reported in the literature [1, 59].”

59.	Moet FJ, Pahan D, Oskam L, Richardus JH; COLEP Study Group. Effectiveness of single dose rifampicin in preventing leprosy in close contacts of patients with newly diagnosed leprosy: cluster randomized controlled trial. BMJ. 2008;336(7647):761–4. doi: 10.1136/bmj.39500.885752.BE.

**With regard to the case characteristics (table 1) there is some unclarity about operational classification and clinical form. PB (26.67%) seems to be the sum of I and TT clinical forms (26.88%), but not just the same. In the sentence starting on line 237 it states: "... there was a predominance of multibacillary cases (73.33%), followed by borderline cases (57.00%)". But borderline cases are part of MB...**

**Authors:** In the databank not all Clinical forms was filled contrary to the Operational classification causing to have disparities between the sums of then. But revising the databank once more we encountered some Operational classification with incompatibility with Clinical form such as Indeterminate being classified as Multibacilary cases thus having to add Not classified/ignored on Operational Classification and put this disparities in this new variable. Finally we re-worked the table based on these new findings. Finally we rewrite the paragraph to make clear for the readers.

“Lines 263-272 Regarding the clinical variables, there was a predominance in operational classification of multibacillary cases (72,8%), and for the clinical form by borderline cases (56,83%). In the data, we encountered some operational classification with incompatibility with clinical form, such as Indeterminate being classified as Multibacilary cases, thus having to add Not classified/ignored on operational classification. Not all clinical forms were filled in the databank contrary to the operational classification, causing disparities between the sum of them. The disability grade at diagnosis showed that 40.19% had G0D, followed by 30.45% that were not evaluated, 21.3% with G1D and 8.06% with G2D.”

**With regard to presentation of trends, I would first like to see the figure 'Detection total' separately with under the time line some bars or arrows indicating important operational changes in time. See for instance the figure A in the following article for inspiration** [**https://www.cell.com/trends/molecular-medicine/fulltext/S1471-4914(20)30065-4**](https://www.cell.com/trends/molecular-medicine/fulltext/S1471-4914(20)30065-4)**. In that way the overall trend is placed in context of the most important operational factors influencing the trend.**

**Authors:** The authors remade the figure accordingly to the suggestion.

**In table 2, make sure to give the full name of the abbreviation AMPC. Furthermore, as reader I need help to understand the importance of the figures under AMPC. Under the heading Trend you state Increasing or Decreasing. But is there a way to indicate a measure of increase or decrease, for instance through a p-value? And how do you establish the importance of a trend. When is the trend such, that you want to highlight it as an important result and discuss its implications. And when is it just some irrelevant 'noise'.**

**Authors:** The authors are grateful for the reviewer's comments and gave the full name in the table 2. In the analysis of time series, we used two techniques to estimate trends: The Seasonal Decomposition of Time Series by Loess (STL) and the Average Monthly Percentage Change (AMPC) technique. The STL technique breaks down the time series into its three components: Trend, Seasonality and Noise. This decomposition occurs through graphics, and for the manuscript, we use only the trend. A limitation of this technique is that it does not provide any measure of increasing or decreasing the trend, consisting only of visual graphs. To try to reduce this limitation, we used the other analysis, AMPC, applying the trends estimated by the STL. This technique provides, on average, an increase or decrease in the trend, providing a percentage measure (%). A limitation of this technique is that it presents only positive (Increasing) and negative (Decreasing) values, not characterizing trends with stationary behavior or any irrelevant noise. Both techniques have limitations (even pointed out by the reviewer) we will mention them in the study's limitations.

**Table 2 summarizes the main findings of the trend figures 2-5. You could therefore consider to place all these figures in an appendix.**

**Authors:** The suggestion was accepted. We included one more figure as a suggestion of an author and changed the names and order of the figures said that; the figures 2, 4-6 is in the appendix.

**Reviewer #5:**

**There is too much background information in the methods – make this more concise, especially the parts about the “study design and research scenario”. Also, in English, we generally don’t say “Research scenario”. I would say “Study Design and Setting”.**

**Authors:** Deleted research scenario e add setting. We are gratefully for the suggestion but we found necessary the background of the city for the complete understanding of the results and also for the suggestions for better coping with this disease that the authors made.

**The paper needs some work before publication, especially in its length, which I think  can be shortened, and in the English / writing in certain places. I found a few typos as well. It needs a thorough review for English language editing, spelling, typos before publication. I also didn't see cover letter or summary which was unusual.**

**Authors:** We shortened the paper as much as possible and it was revised regard the English language, spelling and typos. We also included the considerations of the reviewers. The cover letter was submitted in the PLOs platform. We add the Authors summary in the lines 57-71:

“ In the 2019 report, Brazil had a detection rate of 13,23 per 100,000 inhabitants far from the goal of less than 1 leprosy case per 10,000 inhabitants describe by the World Health Organization. The present study aimed to investigate the epidemiological situation of leprosy and its trend between 2008 and 2018 in a hyperendemic metropolis in the Central-West region of Brazil. A total of 9,739 leprosy cases were reported between 2008 and 2018. The majority of cases were male (58.37%), with a predominant age of 15 to 59 years (87.55%). The predominant level of education was incomplete elementary school (43.96%). The disability grade at diagnosis showed that 40.19% had G0D. There was a predominance in operational classification of multibacillary cases (72,8%). In the trends despite the detection of females and the majority of the ages are decreasing, the detection in males and the disability grade 1,2 and not evaluated in the general population is increasing. Although declining trends were presented, the metropolis is still not close to elimination showing the need prioritize leprosy actions and to improve care for this disease.”

**Line 288 Again, do not say “grade 0 disabilities” are decreasing. That’s misleading. Grade 0 means there is no disability, so you shouldn’t describe it like that. You should say that the rates of new diagnosis without disability is decreasing.**

**Authors:** Rewritten in line 309:

“For all age groups, the rates of new diagnosis with no disability are decreasing…”

**Gender differences – I think you are missing  discussion of a potentially big reason for differences in gender that has been proposed – that women are not seeking care for their leprosy, possibly due to more PB in women than men so underrecognition or possibly due to stigma (reference:  Factors preventing early case detection for women affected by leprosy: a review of the literature. Price VG.Glob Health Action. 2017 Jan-Dec;10(sup2):1360550. doi: 10.1080/16549716.2017.1360550.PMID: 28853325 )**

**Authors:** Complemented in line 511:

“Also, this decreasing trend in women be due to insufficient recognition or stigma [37].”

37. Factors preventing early case detection for women affected by leprosy: a review of the literature. Price VG.Glob Health Action. 2017 Jan-Dec;10(sup2):1360550. doi: 10.1080/16549716.2017.1360550.PMID: 28853325

**There is no discussion on the limitations of this study – one big thing, which you can tell from the figures, is that the number of children with grade 2 disability is actually quite small so it may not be able to tell a clear trend. However, the fact that there are any G2D in children is concerning, and at odds with the WHO goals for zero disability in children < 15 (you should mention these goals in the discussion).**

**Authors:** Rewritten lines 423 and paragraph 537:

“…also the fact that there are any G2D in children is concerning, and it falls far short of the WHO goals for zero disability in children[1, 48, 49].”

1.	WHO 2018. Guidelines for the Diagnosis, Treatment and Prevention of Leprosy. Geneva: World Health Organization; 2018. Licence: CC BY-NC-SA 3.0 IGO. Available from: <https://apps.who.int/iris/handle/10665/274127> (acessed 10 March, 2021).

48.	Xavier MB, Tavares NCS, Corrêa SC, Gonçalves BK, Ramos MAB, Macedo GMM. Correlation between the clinical forms of leprosy and the degree of neurological incapacity. Rev Para Med. 2014; 28(2):15-21. Available from: <http://files.bvs.br/upload/S/0101-5907/2014/v28n2/a4253.pdf> (acessed 10 March, 2021)

49.	Balata ILB. Incapacidade física em menores de 15 anos com diagnóstico de hanseníase [Trabalho de conclusão de curso]. Universidade Federal do Maranhão, Centro de ciências biológicas e da saúde curso de enfermagem; 2018. Available from: <https://monografias.ufma.br/jspui/bitstream/123456789/2066/1/IngridBalata.pdf> (acessed 10 March, 2021)

“A limitation of this study is that the database used is secondary, so it may contain inconsistent information regarding quantity and quality with presence of data that were potentially ignored or incomplete, data regarding the operational classification and Clinical form were, in some cases, not filled completely and**/**or was filled in a dubious way. Another limitation involved STL, which is only a visual resource for showing findings and does not having a "measure" of increase or decrease. AMPC is based on a percentage, which is why does not contain a p-value or 95% confidence interval. The number of children with G2D in the trends is small and thus might not be able to show a clear trend. The interpretation of these data cannot be understood at the individuals’ level.”

**The discussion is too long – you should summarize the different age time series more succinctly and in 1-2 paragraphs and not just repeat your results.**

**Authors** The authors tried to summarize the different ages time series more succinctly.

**I don’t think G0D (as opposed to G1D and G2D for grade 1 and grade2) is an accepted acronym for “no disability” and  it’s confusing to say that G0D is decreasing – G0D in effect is “NO disability”. I would use a different way of describing it because if you read it quickly you may think that some disability is decreasing whereas what you are saying is that people presenting without disability is actually decreasing among many groups which is very concerning.** **For all “G0D”, I would substitute “No disability”**

**Authors:** The authors are grateful for the commentary but in leprosy G0D means grade 0 disability as mentioned in line 186 and is commonly found in several articles [28]. But whenever G0D is mentioned specifically we clarified as suggested with “no disability” for better understanding of the readers.

28.	Srinivas G, Muthuvel T, Lal V, Vaikundanathan K, Schwienhorst-Stich EM, Kasang C. Risk of disability among adult leprosy cases and determinants of delay in diagnosis in five states of India: A case-control study. PLoS Negl Trop Dis. 2019 Jun 27;13(6):e0007495. doi: 10.1371/journal.pntd.0007495. PMID: 31247040; PMCID: PMC6619834

**I know that Asian descent for race translates to “amarelo” in Portuguese, but many English language readers would find “Yellow” offensive, so I would change that to Asian descent.**

**Authors:** We remove all the race/color variable as suggested by the Reviewers.

**Lines 237-238: You wouldn’t really say “followed by borderline cases”, since they are part of two different categories, just state these separately.**

**Author:** We reworked the table and re-write the sentences in line 263-272:

“Regarding the clinical variables, there was a predominance in operational classification of multibacillary cases (72,8%), and for the clinical form by borderline cases (56,83%). In the data, we encountered some operational classification with incompatibility with clinical form, such as Indeterminate being classified as Multibacilary cases, thus having to add Not classified/Ignored on operational classification. Not all clinical forms were filled in the databank contrary to the operational classification, causing disparities between the sum of them. The disability grade at diagnosis showed that 40.19% had G0D, followed by 30.45% that were not evaluated, 21.3% with G1D and 8.06% with G2D.”
