# Supplementary material for: Inequality of gender, age and disabilities due to leprosy and trends in a hyperendemic metropolis: Evidence from an eleven-year time series study in Central-West Brazil
Source: PLoS Negl Trop Dis. 2021 Nov 16;15(11):e0009941. doi: 10.1371/journal.pntd.0009941 (PMC8631739; doi:10.1371/journal.pntd.0009941)
Supplement: Attachment — Submitted filename: Revised Manuscript with Track Changes_corrJHR.docx [file pntd.0009941.s008.docx]

**Inequality of gender, age and disabilities due to leprosy and trends in a hyperendemic metropolis: Evidence from an eleven-year time series study in Central-West Brazil**

José Francisco Martoreli Junior^1*^, Antônio Carlos Vieira Ramos^1^, Josilene Dalia Alves^2^, Juliane de Almeida Crispim^1^, Luana Seles^1^, Thais Zamboni Berra^1^, Tatiana Pestana Barbosa^1^, Fernanda Bruzadelli Paulino da Costa^1^, Yan Mathias Alves^1^, Márcio Souza dos Santos^1^, Dulce Gomes^3^, Mellina Yamamura^4^, Ione Carvalho Pinto^1^ , Miguel Angel Fuentealba Torres^5^, Carla Nunes^6^, Flavia Meneguetti Pieri^7^, Marcos Augusto Moraes Arcoverde^8^, Felipe Lima dos Santos^1^ , Ricardo Alexandre Arcêncio^1^

^1^ Department of Maternal-Infant Nursing and Public Health, University of São Paulo at Ribeirão Preto College of Nursing, Ribeirão Preto, São Paulo, Brazil

^2^ Departament of Epidemiology, Federal University of Mato Grosso, Cuiába, Mato Grosso, Brazil.

^3^ Department of Mathematics, University of Évora, Évora, Portugal.

^4^Departament of Nursing, Federal University of São Carlos, São Carlos, São Paulo, Brazil.

^5^Faculty of Nursing and Obstetrics, University of los Andes, Santiago, Chile.

^6^Department of Public Health, New University of Lisbon, Lisbon, Portugal.

^7^ Department of Nursing, Londrina State University, Londrina, Paraná, Brazil.

^8^ Center for Education, Letters and Health, Western Paraná State University, Campus Foz do Iguaçu, Foz do Iguaçu, Paraná, Brazil.

***** Corresponding author

E-mail: jose.martoreli@usp.br

**ABSTRACT**

The present study aimed to investigate the epidemiological situation of leprosy, in a hyperendemic metropolis in the Central-West region of Brazil. We studied trends over eleven years, both in the detection of the disease and in disabilities, analyzing disparities and/or differences regarding gender and age. This is an ecological time series study conducted in Cuiabá, capital of the state of Mato Grosso. The population consisted of patients diagnosed with leprosy between the years 2008 and 2018. Descriptive statistics were applied, with the calculation of absolute frequencies (*n*) and relative frequencies (%). The time series of leprosy cases was used, stratifying it according to gender (male and female), grade of disability (G0D, G1D, G2D, and not evaluated) and age. The calendar adjustment technique was applied, taking into account the number of days of each month in the calculation. For modeling the trends, the Seasonal-Trend decomposition procedure based on Loess (STL) was used. We identified 9,739 diagnosed cases, in which 58.37% were male and 87.55% aged between 15 and 59 years. Regarding detection according to gender, there was a decrease among women and an increase in men. There was a trend towards an increase in disabilities in both genders. The study advances the knowledge by demonstrating an increasing trend in disabilities in both genders, which may be related to the delay in diagnosis. There was also an increasing number of cases that were not assessed for disability at the time of diagnosis, which denotes the quality of the services.

**INTRODUCTION**

Leprosy is a chronic infectious disease caused by the bacillus *Mycobacterium leprae*, which affects Schwann cells, causing their destruction, affecting the skin, and resulting in severe neuropathies, which can lead to physical disabilities. It is believed that its transmission takes place through droplets; however, other means cannot be excluded [1]. In the past 30 years, the World Health Organization (WHO) has sought measures to eliminate leprosy, and although its indicators have been decreasing over the years, the goal of elimination (prevalence <1 case per 10,000 inhabitants) has not yet been reached and currently seems to be more distant than imagined [2,3].

According to WHO data in the 2019 report, Brazil, India and Indonesia reported >10,000 new cases, classifying them as the three most highly endemic countries [4]. In 2019 Brazil had a detection rate of 13,23 per 100,000 inhabitants [5].. It is the country with the second highest number of leprosy occurrences only behind India, and it is the country with the most cases in the Americas [6].

According to the “Global Leprosy Strategy 2016−2020: Accelerating towards a leprosy-free world” [1], the main actions to control the disease should be directed toward early detection, reduction in the number of cases, and reduction of cases with deformities, mainly in children. In addition, there should be a reduction in the time of diagnosis, preventing cases with disabilities. It is also expected that the services will systematically assess their diagnosed cases regarding disabilities in a longitudinal manner throughout the treatment, thus avoiding more severe sequelae. The strategy reinforces the prevention and management of physical disabilities by interprofessional teams, the search for contacts, careful and planned monitoring of cases already diagnosed, and the reduction of stigma, a major complication in seeking services and treatment adherence [6-8].

Brazil is a country of continental proportions, with an extensive land border, neighboring 10 of the 12 other countries in South America, which makes leprosy control a major challenge. It is divided into five macro-regions, among which the Central-West is one of the most problematic regions in terms of the burden of the disease. A study carried out in the region showed that in the trienniums of 2001 - 2003 and 2010 -2012 there was a reduction in the disease; however, there are geographical areas where leprosy control has not advanced and are far from elimination [9]. The official reports from Brazil indicate the reduction of leprosy in general, but there is evidence that there is gender inequity in access to health services, which may have implications for the disease [10]. Another issue refers to age, as there is evidence that the population aging process is changing the profile of the leprosy morbidity profile [11], given the number of people falling ill in a context of poverty and inequality, with older adults having more difficulty in accessing health services and tending to have a more unfavorable prognosis. This needs to be better addressed from the perspective of health surveillance.

Also, regarding the inequality related to age, it is known that when there is a delay in diagnosis, children who had contact with index cases also become ill [12], which is an important gap to be filled. Accordingly, the elimination of leprosy involves comprehending the determinants, according to a gender and age equity perspective. It is also understood that gender and age inequity should not be analyzed only from the perspective of confirmed detection and/or diagnosis, but also in terms of disability, as the WHO recognizes the disability grade indicator as the most sensitive measure of the real leprosy situation in a community and because we cannot count “undetected cases”, expressing, therefore, the fragility of the services in detecting the disease early [1].

There are hypotheses that there are gender differences, and that older adults and children are more severely affected by the disease, with regard to disabilities, and this will continue in the coming years. These aspects need to be studied in order to define public health policies and plan strategic actions in priority areas, as well as to advance equity. There are several tools that could be used to test these hypotheses; among them, one of the more sensitive tools is the time series. Its use is justified in the field of public health, as it can show the behavior of the disease in vulnerable groups and verify how much success has been achieved in terms of the goal of elimination and reduction of injustices and/or inequity. Although time series studies have been exhaustively applied to identify the dynamics of COVID-19 and even make predictions, supporting decisions regarding lockdowns and/or social isolation [13,14], there are few studies on leprosy that have this intention [15].

This study aimed to investigate the epidemiological situation of leprosy and trends in the detection of cases and disabilities, and to evidence disparities and/or differences regarding gender and age in a hyperendemic metropolis in Central-West Brazil.

**MATERIALS AND METHODS**

**Study design and setting**

This ecological time series study [16] was carried out in Cuiabá, capital of the state of Mato Grosso, located in the Central-West region of Brazil (Fig 1). The metropolis has an area of 3,266,538 km^2^, with an estimated population of 607,153 inhabitants and demographic density of around 185.87 inhabitants per km^2^ in 2018 [17].

**Fig 1. Location of Cuiabá - Mato Grosso.** (A) Brazil; (B) State of Mato Grosso; (C) City of Cuiabá

In relation to socioeconomic indicators, Cuiabá has an illiteracy rate of 4.56% for women and 4.79% for men, a life expectancy at birth of 75 years of age, and a Human Development Index (HDI) of 0.785. It also has a Gini index of 0.59, an indicator that measures how equitably a resource is distributed in a population; the nearest to 0 is the population with lowest inequality, and closest to 1 is the most unequal [18]. Regarding basic sanitation, 53.52% of Cuiabá’s territory has a sewage network and 98.12% a water supply [19,20]. It also has 63 Primary Health Units, 4 Polyclinics, and 2 Emergency Care Units, distributed throughout 4 Administrative Regions north, south, east, and west, which have variable numbers of neighborhoods [21,22]. It should be highlighted that the municipality provides the following referral services for procedures of greater technological complexity as well as for leprosy, CERMAC - (dermatology surveillance services), metropolitano hospital (hospital care for leprosy surgery services), Julio Muller university hospital (hospital care for ophthalmology referral), CRIDAC center specialized in rehabilitation (CER III), and regional outpatient and hospital referrals [23,24].

The municipality presents a detection rate of 45.30 cases for every 100,000 inhabitants, classifying it as hyper endemic for leprosy according to the Notifiable Disease Information System (SINAN) 2018, which classifies hyper endemic cities with a detection greater than 40 cases for every 100,000 [25,26]. In children under 15, in 2018 the detection rate was 7.9/100,000 inhabitants, placing the metropolis in the very high category for children. Regarding the disability grade, health units evaluated a mean of 56.24% of new cases at the time of diagnosis, below expectations, which should be at least 75.00% according to SINAN [25, 26].

**Study population and information sources**

Leprosy cases registered in the SINAN from 2008 to 2018 of only residents of the city of Cuiabá were included. The SINAN is the Brazilian information system responsible for recording and processing information on mandatory notifiable diseases such as leprosy throughout Brazil, providing bulletins and reports of morbidity and constituting one of the main surveillance systems in the country.

According to the Brazil Practice Guide for Leprosy, the cases are diagnosed if the person presents a defined area with altered or completely lost of sensitivity with or without compromised nerve, or nerves with neural thickening, and/or positive bacilloscopy, the bacilloscopy is not the defining factor but always the clinical and epidemiological evaluation [27].

The selected variables were date of notification of the case, gender (male, female), age, education (no schooling, incomplete elementary education, complete elementary education, incomplete high school education, complete high school education, incomplete higher education and complete higher education), WHO operational classification (Paucibacillary, Multibacillary), clinical form based on Madrid classification (indeterminate, tuberculoid, borderline and lepromatous) and assessment of disability grade in the diagnosis (Grade 0 disability [G0D], Grade 1 disability [G1D], Grade 2 disability [G2D], and not evaluated) [28].

**Statistical analysis**

Initially, the variables of interest were standardized, with data relating to age, gender, and education considered in the sociodemographic dimension and operational classification, clinical form, and d disability grade at diagnosis in the clinical-epidemiological dimension. Descriptive analysis of the sociodemographic and clinical-epidemiological variables was performed with the calculation of absolute frequencies (*n*) and relative frequencies (%).

Next, the time series of leprosy cases were constructed according to the total number of cases, gender (male and female), and disability grade (G0D, G1D, G2D, and not evaluated). Time series correspond to the set of observations ordered in time and recorded at regular periods, the aim of which is to identify patterns (increase, decrease, stationary) in a variable of interest over time [29,30]. For the construction of the time series, the general rates of detection and those stratified by gender were calculated, considering the total population of the municipality (for general detection rate) and the populations of men and women (for rates stratified according to gender) as the denominator, all with a multiplication factor per 100,000 inhabitants. For the construction of time series of the cases with disability grade we considered all the number of cases with disability grade (G0D, G1D, G2D, and not evaluated).

After the construction of the time series, according to detection in general, by gender, age, and disability grade, the evolutions of the trends were calculated using the Seasonal-Trend decomposition procedure based on Loess (STL) [31]. This methodology is based on the classic decomposition of time series that disaggregates the total series into three additive components (trend, seasonality or error), allowing each of these components to be separately estimated and identifying the source of variability of the series in a more concise way than through a global analysis of the series. The STL has a simple design that consists of a sequence of applications of the Loess smoother and allows analysis of the properties of the procedure and quick calculations even for a very long time series and large amounts of trend and seasonal smoothing, a very small amount of trend smoothing, and seasonal components that are not distorted by aberrant behavior in the data ability to decompose time series with missing values. [32].

One of the advantages of this methodology is that it is quite robust regarding the existence of outliers (present in the series, Figs .2 -6). Trend refers to the general direction in which the variables of the time series develop, according to a time interval, presenting a pattern of increase/decrease of the variable over a certain period. Seasonality is identical patterns that a time series seems to obey and that occur regularly at fixed periods of time. Finally, noise is the fluctuations that occur over the time of the series, being irregular and random movements perceptible only with the removal of the other components [33].

Having estimated the three components of the time series, only the trend was selected to characterize the behavior of the variables of interest over time. Subsequently, the Average Monthly Percentage Change (AMPC) was calculated for the trends in the general detection rates by gender and cases with disability grade identifying the mean percentages and how much the trends increased or decreased over the study period. Finally, the trend in the rate of detection of disability grade at the leprosy diagnosis and the trend of each disability grade, both by age group, were also analyzed. All analyses were performed using the R Studio® version 3.5.2 statistical software.

Finally, we mention that the notification forms are filled out by third parties, so dubious data may be provided.

**Ethical aspects**

The study was approved by the Ethics Committee of the University of São Paulo, School of Nursing (EERP/USP) under CAAE: 30394720.3.0000.5393. The investigation was exempted from the signing of consent forms, as it used secondary data, considering that the data were analyzed in an aggregated manner, without individual identification.

**RESULTS**

A total of 9,739 leprosy cases were reported between 2008 and 2018. As shown in Table 1, the majority of cases were male (58.37%), with a predominant age of 15 to 59 years (87.55%). The predominant level of education was incomplete elementary school (43.96%).

**Table 1 - Clinical and social epidemiological characteristics of the cases diagnosed with leprosy, in an endemic municipality in Central-West Brazil (2008-2018)**

| **Variables** | **Frequency (*n*=9739)** | **%** |
| --- | --- | --- |
| **Gender** |  |  |
| Male | 5685 | 58.37 |
| Female | 4052 | 41.60 |
| Did not answer | 2 | 0.03 |
| **Age** |  |  |
| <15 years | 514 | 5.28 |
| 15 to 59 years | 8527 | 87.55 |
| 60 years or more | 698 | 7.17 |
| **Education** |  |  |
| No schooling | 738 | 7.58 |
| Incomplete elementary education | 4279 | 43.96 |
| Complete elementary education | 821 | 8.43 |
| Incomplete High School Education | 736 | 7.55 |
| Complete high school education | 1621 | 16.64 |
| Incomplete higher education | 262 | 2.70 |
| Complete higher education | 552 | 5.65 |
| Did not answer | 650 | 6.67 |
| Not applicable | 80 | 0.82 |
| **Operational classification** |  |  |
| Paucibacillary | 2582 | 26.5 |
| Multibacillary  Not classified/ignored | 7095  62 | 72.8X  0.7X |
| **Clinical form** |  |  |
| Indeterminate | 1118 | 11,48 |
| Tuberculoid | 1456 | 14,95 |
| Borderline | 5535 | 56,83 |
| Lepromatous | 1422 | 14,60 |
| Not classified/ignored | 208 | 2,14 |
| **Disability grade at diagnosis** |  |  |
| Grade 0 | 3914 | 40.19 |
| Grade I | 2074 | 21.30 |
| Grade II | 785 | 8.06 |
| Not evaluated | 2966 | 30.45 |

.

Regarding the clinical variables, there was a predominance in operational classification of multibacillary cases (72.8x%), and for the clinical form by borderline cases (56.83%). In the data, we encountered some operational classification with incompatibility with clinical form, such as Indeterminate being classified as Multibacillary cases, thus having to add Not classified/Ignored on operational classification. Not all clinical forms were filled in the databank contrary to the operational classification, causing disparities between the sum of them. The disability grade at diagnosis showed that 40.19% had G0D, followed by 30.45% that were not evaluated, 21.3% with G1D and 8.06% with G2D.

**Table 2 and Figs 2 -6 present the trends in detection and disability grade rates**.

According to the results, for the detection rates (Fig2 in S3 Figure) there was a decreasing trend in the general detection rate and for the female gender, and an increasing trend for the male gender (0.01%). Considering disability grade in the general population (Fig4 in S4 Figure) , no disability (G0D) was the only grade with a decreasing trend, while G1D (0.56%), G2D (0.38%), and unevaluated cases (0.28%) showed increasing tendencies.

**Table 2 - Mean percentage variation in the rates of detection of leprosy and Grade of Disability at the time of diagnosis of cases, in an endemic municipality in Central-West Brazil (2008–2018)**

| **Variables** | **Average Monthly Percentage Change (AMPC) (%)** | **Trend*** |
| --- | --- | --- |
| **LEPROSY DETECTION** |  |  |
| General population | - 0.11 | Decreasing |
| **Gender** |  |  |
| Male | 0.01 | Increasing |
| Female | - 0.26 | Decreasing |
| **Age group** |  |  |
| <15 years | -0.99 | Decreasing |
| 15 – 29 | -0.49 | Decreasing |
| 30 – 59 | 0.07 | Increasing |
| Over 60 | -0.0028 | Decreasing |
| **DISABILITIES** |  |  |
| **Disability grade in the general population** |  |  |
| Grade 0 | -0.57 | Decreasing |
| Grade 1 | 0.56 | Increasing |
| Grade 2 | 0.38 | Increasing |
| Not evaluated | 0.28 | Increasing |
| **Disability grade in males** |  |  |
| Grade 0 | -0.44 | Decreasing |
| Grade 1 | 0.35 | Increasing |
| Grade 2 | 0.67 | Increasing |
| Not evaluated | 0.22 | Increasing |
| **Disability grade in females** |  |  |
| Grade 0 | -0.7 | Decreasing |
| Grade 1 | 1.24 | Increasing |
| Grade 2 | -6.09 | Decreasing |
| Not evaluated | 0.45 | Increasing |
| **Disability grade in children under 15** |  |  |
| Grade 0 | -0.26 | Decreasing |
| Grade 1 | 1.02 | Increasing |
| Grade 2 | 9.86 | Increasing |
| Not evaluated | -0.42 | Decreasing |
| **Disability grade in people aged 15 to 29 years** |  |  |
| Grade 0 | -0.56 | Decreasing |
| Grade 1 | 0.98 | Increasing |
| Grade 2 | 0.04 | Increasing |
| Not evaluated | -0.91 | Decreasing |
| **Disability grade in people aged 30 to 59** |  |  |
| Grade 0 | -0.47 | Decreasing |
| Grade 1 | 0.74 | Increasing |
| Grade 2 | -0.62 | Decreasing |
| Not evaluated | 0.63 | Increasing |
| **Disability grade in people aged 60 years or more** |  |  |
| Grade 0 | -0.40 | Decreasing |
| Grade 1 | 0.73 | Increasing |
| Grade 2 | 0.67 | Increasing |
| Not evaluated | 0.21 | Increasing |

Source: Authors. * Considered the mean, which is influenced by changes and/or extreme variations.

Fig 3 presents the main changes in the structure of the time series of the total detection rate of leprosy cases. Three changes of structure are verified in the series; the first occurred in November 2011, in which the series shows a marked decrease until the year 2013. The second change in structure occurred in August 2013, with an increase in the time series, presenting the highest values in the years 2014 and 2015, with a detection rate of more than 20 cases per 100,000 inhabitants. As of October 2015, the last change in structure occurred, with a decrease and later stability in the detection rate values until the end of the series.

**Fig 3. Changes in the structure of the time series for the general leprosy detection rate, Cuiabá, Mato Grosso, Brazil (2008–2018).** (A) Time series; (B) Trend; (C) Change of structure; (D) Point of structural change.

When stratifying Grade of Disability according to gender (Fig5 in S5 Figure)there was a decreasing trend for no disability (G0D) in males (-0.44%) and females (-0.70%), and a decrease in G2D in females (-6.09%). The other Grade of Disability showed increasing trends throughout the series.

In Figure 6 (Fig6 in S6 Figure) shows the Grade of Disability according to age groups. For all age groups, the rates of new diagnosis with no disability (G0D) are decreasing. Regarding G1D, all age groups showed an increasing trend. For G2D, only the 30**–**59 years age group presented a decreasing trend (-0.62), with the other groups showing increasing trends. Finally, regarding those not evaluated for Grade of Disability, children aged under 15 years (-0.42) and the group aged 15 to 29 years (-0.91) showed decreasing trends, while the other groups (30 to 59 years and 60 years or more) presented increasing trends.

**DISCUSSION**

The present study aimed to investigate the epidemiological situation of leprosy, its trend over the years, and whether there were trends, both in the detection of the disease and in disabilities, analyzing disparities and/or differences regarding gender and age in a hyperendemic metropolis in the Central-West region of Brazil. The study showed that leprosy has been declining in the scenario under study; however, when analyzed according to gender, we observed that the detection was increasing in men but decreasing in women, which shows gender inequity. The detection rate was also found to be increasing in the adult and economically active population: however, it was decreasing in children and adolescents (under 15 years of age) and in older adults (aged 60 years or more). Considering disabilities, it was found that individuals without disabilities (G0D) were decreasing, but individuals with disabilities (G1D and G2D) were increasing, which raises the hypothesis of late diagnosis and/or underreporting.

Literature shows that the appearance of physical disabilities in people affected by leprosy is an indication of late diagnosis [34]. The fact that the most serious cases occurred in children, young adults and older adults, demonstrates weaknesses of the health services in the early detection of these cases and also the lack of knowledge of the population about the signs and symptoms suggestive of the disease, ignorance on the part of health care providers, difficulties related to early diagnosis, and/or the silent evolution of the disease. An important result was the significant reduction in cases without disabilities (G0D), as well as an increasing trend in cases where disability was not evaluated at the time of diagnosis, once again revealing a weakness of the health services. These services should address the stigma and discrimination that continues to hinder the spontaneous seeking of a diagnosis and treatment for the disease [35]. These disabilities can lead to permanent sequelae, as they result from injuries to the peripheral nerves responsible for the sensation of pain, touch, and heat, in addition to the possible impairment of the vision of the person affected, making the individual susceptible to accidents and injuries [36].

A current ally to fight leprosy is leprosy post-exposure prophylaxis (LPEP). According to the WHO guidelines, single-dose rifampicin (SDR) can be used in children and adults [1]. In a randomized controlled trial, SDR given to leprosy contacts provided a reduction in leprosy risk of 57% in 2 years and 30% in 5-6 years [37]. As leprosy is a highly stigmatized disease, care should be taken to reveal the identity of the index patient when implementing this preventive therapy in contacts, especially outside the patient's family.

The result that men are more affected than women has also been found in other studies [38,39]. This can be related to several factors, such as less concern about their own health and difficulties for men to access public health services [40,41]. The male population’s lack of self-care has been recorded in the literature since the 1990s [42].Currently, there are few health policies aimed at this population to meet their needs [43]. Barriers related to the difficulty of access to health services, the incompatibility between the hours of operation of health units and the workday, and the belief of less susceptibility in relation to women contribute to this greater burden of leprosy in the male population [44].

Most of the cases had incomplete elementary education, an indicator of low schooling, which may be related to the social aspect and living conditions. Low levels of education hinder access to better jobs and better economic conditions [45,46].

The predominance of multibacillary cases, with the most severe clinical forms (especially borderline and lepromatous cases), may suggest the occurrence of active transmission of the disease and, consequently, greater potential to incapacitate the affected individuals [47].

In the state of Mato Grosso in 2015, the “National Campaign for Leprosy, Geohelminthiasis and Trachoma” was initiated, which mobilized local health services to execute actions related to the active search for cases, focusing on schoolchildren aged 5 to 14 years. The campaign was carried out in approximately 915 schools in 65 municipalities (including Cuiabá), to examine and treat more than 291.200 students and their possible contacts [24]. It is estimated that the campaign may have had an impact in the region studied, reflecting the peak of detection verified in the study for this age group. No further policies were encountered in public archives to influence the detection of leprosy in the region durinh the time period.

In relation to children under 15 years of age, when analyzing the Grade of Disability, there were growing trends in the number of G1D and G2D in this age group, which may indicate that the municipality faces difficulties in the early diagnosis of the disease. A study by Xavier et al. (2014) with children under 15 years of age indicated that the early exposure to the pathogen in this age group suggests a late diagnosis and prolonged exposure [48]. People who are affected by multibacillary forms of the disease also have a greater chance of developing health problems and the fact that there are any G2D in children is concerning, as it falls far short of the WHO goal for zero disability in children [1, 48, 49]. Leprosy is highly disabling when not properly treated in this population, which can influence school performance and cause problems related to social limitations, self-esteem, and stigma experienced by the affected person, especially because this is a period of growth and physical and emotional development [50]. This age group is indicative of foci of transmission of recent infection, due to the characteristic of the disease being transmitted by lengthy exposure to the bacillus without adequate treatment [51].

Regarding the age group of 15 to 29 years of age, despite presenting a decreasing trend in the case detection rate, it should be noted that G1D and G2D ended the series with increasing trends. These results can be strong indications of late diagnosis and underreporting of cases [47,52], since the decrease in the detection rate of new cases is not accompanied by the decrease of cases with Grade of Disability, considering that these cases, especially G2D, are indications of active transmission of the disease, contradicting the results related to the detection of new cases. This constitutes a warning about possible difficulties of health services in detecting and conducting the search for these cases early. A study carried out in the state of Paraíba observed that in the period from 2006 to 2016, the detection of leprosy was only made after referral to specialized services, without active search actions in the population, making early diagnosis even more difficult [53].

The group aged 30 to 59 years ended the time series with an increasing trend in the detection rate of new cases, as well as in G1D. This age group is composed of the economically active population, where the disabilities and incapacities caused by leprosy affect the work and social life environments, causing not only economic losses for the individual and his or her community but also psychological losses [9,54,55]. These findings also corroborate other studies, which show the same problems, with the interruption of employment relations directly affecting the quality of life [1,5]. The older adult age group (aged 60 years or more) had a decreasing trend in the rate of detection of new cases and increasing values in G1D and G2D. The antagonism between the decrease in the detection rate and the increase in disabilities is a strong indication of difficulties in the active search for cases and the possibility of underreporting [43,52].

The issue of not evaluating cases for disability showed an increasing trend over the study period. The assessment of physical disability at the time of diagnosis is a priority action for newly diagnosed leprosy cases, and the fact that “non-evaluation” presents an increasing trend may raise discussions regarding the management of leprosy cases in the research setting.These findings lead us to suppose that the assessment and registration at the health care level/national surveillance system of people affected by the disease had not been carried out systematically, with the number of unevaluated cases often showing a lack of adherence, unpreparedness, and a lack of standardized protocols that guide the classification and registration, of both the leprosy and its disability grade. This was reflected in the patterns found in the present study, which show that, despite declining trends, the metropolis is still not close to elimination [6].

Leprosy affects men and women, age groups and social classes in different ways, it emerges from inequality and produces more inequality among the affected populations. In addition to this discussion, it can also be defined as neglected, both by public policies and health authorities, with regards to incorporating it into priority investment actions, considering that it affects the most marginalized populations and those in situations of extreme poverty. Its incidence in these populations contributes to the worsening of the situation of extreme poverty, accentuating the vulnerabilities and the ailments resulting from injustices [56-58].

This is the first study that sought to comprehend the situation of leprosy according to gender and age, showing that the disease trend is not homogeneous among groups, but seriously affects more vulnerable people. The findings show that the disease is growing in men, with this gender being most affected by more severe disabilities, which denotes its difficulty in accessing health services. The detection of leprosy in women has also been decreasing; however, the number of disabilities, although on a smaller scale than in men, has been increasing, indicating the need for different approaches for the genders, in both promotion actions and the diagnosis as awareness raising by the city’s health service and Primary Health Units. Also, this decreasing trend in women be due to insufficient recognition or stigma [37].

The findings also highlighted another issue of concern, namely the growing number of disabilities in children and older adults. These segments should be priorities for policies and social protection actions by the state, given that in these stages of life people are in situations of vulnerability, therefore requiring state support. In general, the studies carried out on leprosy, including the WHO reports, sought to generally analyze the trend of the disease in the general population, always showing a decrease in the disease as a result, which was confirmed in the present study[1,4,15]. This result can also be caused by a lack of active case-finding activities, thus not always leading to a positive finding [1,4,15]. However, when stratifying by vulnerable segments, we still observe great inequalities and disparities in the context of leprosy.

If there is a clear intention to overcome leprosy, investments in health and different methodological approaches must be considered, in both research and the surveillance system of health systems and territories. The elimination of leprosy involves comprehending the differences in gender issues and life cycles in the health territories, and the elaboration of intervention projects aimed at overcoming the problem must be sufficiently supported by evidence from these approaches. Guiding public policies based on this evidence is essential for advancing equity and eliminating leprosy. The health system that determines strategic actions for leprosy in this region could focus on actions for the health care providers to correctly evaluate the disabilities. Active search actions can promote the new detection of cases, and the search and follow-up of their communicators must also be promoted. As was mentioned, LPEP and SDR could be studied by managers to implement them based on the WHO’s recommendations and their effectiveness as reported in the literature [1, 59].A limitation of this study is that the database used is secondary, so it may contain inconsistent information regarding quantity and quality with presence of data that were potentially ignored or incomplete, data regarding the operational classification and Clinical form were, in some cases, not filled completely and**/**or was filled in a dubious way. Another limitation involved STL, which is only a visual resource for showing findings and does not having a "measure" of increase or decrease. AMPC is based on a percentage, which is why does not contain a p-value or 95% confidence interval. The number of children with G2D in the trends is small and thus might not be able to show a clear trend. The interpretation of these data cannot be understood at the individuals’ level.

In conclusion, the present study highlights the need to prioritize leprosy actions and to improve care for this disease, as well as to develop strategies for early detection and its more effective insertion into healthcare networks.

**Acknowledgments**

The authors would like to thank the Health Surveillance Service of the Cuiabá Regional Health Management Unit, of the state government of Mato Grosso for making the data available, the CNPQ research productivity grant (process no. 304483/2018-4). This study was financed in part by the Coordenação de Aperfeiçoamento de Pessoal de Nível Superior – Brasil (CAPES) – Finance Code 001.

**References**

1.	WHO 2018. Guidelines for the Diagnosis, Treatment and Prevention of Leprosy. Geneva: World Health Organization; 2018. Licence: CC BY-NC-SA 3.0 IGO. Available from: <https://apps.who.int/iris/handle/10665/274127> (acessed 10 March, 2021).

2.	Barbosa CC, Bonfim CV, Brito CMG, Ferreira AT, Gregório VRN, Oliveira ALS, et al. Spatial analysis of reported new cases and local risk of leprosy in hyper-endemic situation in Northeastern Brazil. Trop Med Int Heal. 2018; 23(7):748-757. Available from: <https://doi.org/10.1111/tmi.13067> PMID: 29704447

3.	Mitjà O, Marks M, Bertran L, Kollie K, Argaw D, Fahal AH, et al. Integrated Control and Management of Neglected Tropical Skin Diseases. PLoSNegl Trop Dis. 2017; 11(1):e0005136. Available from: <https://doi.org/10.1371/journal.pntd.0005136> PMID: 28103250

4.	WHO 2020 Weekly epidemiological record Global leprosy (Hansen disease) update, 2019: time to step-up prevention initiatives 4 SEPTEMBER 2020, 95th YEAR / 4 SEPTEMBRE 2020, 95e ANNÉE No 36, 2020, 95, 417–440 Available from: https://www.who.int/publications/i/item/who-wer9536

5.	Ministério da Saúde Secretaria de Vigilância em Saúde Hanseníase 2021 Available from: <https://www.gov.br/saude/pt-br/media/pdf/2021/fevereiro/12/boletim-hanseniase-_-25-01.pdf>

6.	WHO 2016. Global Leprosy Strategy 2016–2020: Accelerating towards a leprosy-free world. Geneva: WHO; 2016. Licence: CC BY-NC-SA 3.0 IGO. Available from: <https://apps.who.int/iris/handle/10665/254907> (acessed 10 March, 2021)

7.	Brasil. Ministério da Saúde. Secretaria de vigilância em saúde. Departamento de vigilância das doenças transmissíveis. Diretrizes para vigilância, atenção e eliminação da Hanseníase como problema de saúde pública: manual técnico-operacional. Brasília: Ministério da Saúde; 2016. Available from: <https://portalarquivos2.saude.gov.br/images/pdf/2016/fevereiro/04/diretrizes-eliminacao-hanseniase-4fev16-web.pdf> (acessed 10 March, 2021).

8.	Brasil. Ministério da Saúde. Secretaria de Vigilância em Saúde. Departamento de Vigilância Epidemiológica. Manual de Prevenção de Incapacidades – Série A Normas e Manuais Técnicos. Brasília: Ministério da Saúde; 2008. Available from: <http://bvsms.saude.gov.br/bvs/publicacoes/manual_prevencao_incapacidades.pdf> (acessed 10 March, 2021).

9.	Freitas RS, Duarte EC, Garcia LP. Analysis of the epidemiological situation of leprosy in an endemic area in Brazil: spatial distribution in the periods 2001 – 2003 and 2010 – 2012. Rev Bras Epidemiol. 2017; 4(20):702-713. Available from: <http://dx.doi.org/10.1590/1980-5497201700040012>

10.	Barbosa EC, Cookson R. Multiple inequity in health care: An example from Brazil. Soc Sci Med. 2019; 228:1-8. Available from: <http://dx.doi.org/10.1016/j.socscimed.2019.02.034> PMID: 30856368

11.	Oliveira JSS, Reis ALMD, Margalho LP, Lopes GL, Silva ARD, Moraes NS, et al. Leprosy in elderly people and the profile of a retrospective cohort in an endemic region of the Brazilian Amazon. PLoS Negl Trop Dis. 2019; 13(9):e0007709. Available from: <http://dx.doi.org/10.1371/journal.pntd.0007709> PMID: 31479442

12.	Vieira MCA, Nery JS, Paixão ES, Andrade KVF, Penna GO, Teixeira MG. Leprosy in children under 15 years of age in Brazil: A systematic review of the literature. PLoS Negl Trop Dis. 2018; 12(10):e0006788. Available from: <http://dx.doi.org/10.1371/journal.pntd.0006788> PMID: 30278054

13.	Ismail L, Materwala H, Znati T, Turaev S, Khan MAB. Tailoring time series models for forecasting coronavirus spread: Case studies of 187 countries. Comput Struct Biotechnol J. 2020; 18:2972-3206. Available from: <http://dx.doi.org/10.1016/j.csbj.2020.09.015> PMID: 32994886

14.	Merow C, Urban MC. Seasonality and uncertainty in global COVID-19 growth rates. Proc Natl Acad Sci USA. 2020; 117(44):27456-27464. Available from: <http://dx.doi.org/10.1073/pnas.2008590117> PMID: 33051302

15.	Ramos ACV, Gomes D, Neto MS, Berra TZ, Assis IS, Yamamura M, et al. Trends and forecasts of leprosy for a hyperendemic city from Brazil’s northeast: Evidence from an eleven-year time-series analysis. PLoS One. 2020; 15(8):e0237165. Available from: <http://dx.doi.org/10.1371/journal.pone.0237165> PMID: 32764785

16.	Morgenstern H. Ecologic studies in epidemiology: concepts, principles, and methods. Annu Rev Public Health. 1995; 16:61-81. Available from: <http://dx.doi.org/10.1146/annurev.pu.16.050195.000425> PMID: 7639884

17. Instituto Brasileiro de Geografia e Estatística (IBGE). IBGE cidades/Panorama/Cuiabá/MT. 2019. Available from: <https://cidades.ibge.gov.br/brasil/mt/cuiaba/panorama> (acessed 10 March, 2021)

18.	Frank A. Farris (2010) The Gini Index and Measures of Inequality, The American Mathematical Monthly, 117:10, 851-864, DOI: [10.4169/000298910X523344](https://doi.org/10.4169/000298910X523344)

19.	Brasil. Ministério do Desenvolvimento Regional. Ranking ABES da Universalização do Saneamento. Brasília: Sistema Nacional de Informações sobre Saneamento, 2019. Available from: <http://abes-dn.org.br/pdf/Ranking_2019.pdf> (acessed 10 March, 2021)

20.	Brasil. Programa das Nações Unidas para o Desenvolvimento. Atlas Do Desenvolvimento Humano No Brasil, Fundação João Pinheiro, Governo do estado de Minas Gerais; 2018.

21.	Brasil. Prefeitura Municipal de Cuiabá. Secretaria de Saúde. Unidades Básicas de Saúde. Cuiabá; 2019.

22.	Brasil. Prefeitura Municipal de Cuiabá. Secretaria de Saúde. Unidades De Pronto Atendimento - Policlínicas E Up’s; 2019.

23.	Brasil. Prefeitura Municipal de Cuiabá. Instituto de Planejamento e Desenvolvimento Urbano. Organização Geopolítica de Cuiabá; 2007.

24.	Brasil. Secretaria do Estado de Saúde - SES – MT. Assessoria da secretaria de saúde do estado do Mato Grosso. Campanha contra hanseníase, verminoses e tracoma termina dia 29. Brasília: SES-MT; 2015. Available from: <http://www.mt.gov.br/-/campanha-contra-hanseniase-verminoses-e-tracoma-termina-dia-29> (acessed 10 March, 2021)

25.	Brasil. Prefeitura de Cuiabá. Boletim Epidemiológico Hanseníase. Cuiabá: SES; 2019. Available from: <http://201.24.3.67:8080/portal/upload/arquivos/20190523165956000163.pdf> (acessed 10 March, 2021)

26.	MINISTÉRIO DA SAÚDE, Roteiro para uso do Sistema de Informação de Agravos de Notificação – Sinan NET para hanseníase 2018. Available from: <http://portalsinan.saude.gov.br/images/documentos/Agravos/Hanseniase/Manual_tabulacao_dos_indicadores_de_hanseniase.pdf>

27.	MINISTÉRIO DA SAÚDE, Guia prático sobre a Hanseníase – Secretaria de Vigilância em Saúde – Departamento de Vigilância e Doenças Transmissíveis - Brasília - DF 2017 Availble from: https://portalarquivos2.saude.gov.br/images/pdf/2017/novembro/22/Guia-Pratico-de-Hanseniase-WEB.pdf

28.	Srinivas G, Muthuvel T, Lal V, Vaikundanathan K, Schwienhorst-Stich EM, Kasang C. Risk of disability among adult leprosy cases and determinants of delay in diagnosis in five states of India: A case-control study. PLoS Negl Trop Dis. 2019 Jun 27;13(6):e0007495. doi: 10.1371/journal.pntd.0007495. PMID: 31247040; PMCID: PMC6619834

29.	Latorre MRDO, Cardoso MRA. Time series analysis in epidemiology: an introduction to methodological aspects. Rev bras epidemiol. 2001; 4(3):145–152. Available from: <https://doi.org/10.1590/S1415-790X2001000300002>

30.	Cardoso JA. Limitação de atividade funcional e participação social em hanseníase: fatores associados em área hiperendêmica do nordeste brasileiro [dissertação]. Universidade Federal do Piauí, Centro de Ciências da Saúde/Departamento de Enfermagem; 2020. Available from: <https://repositorio.ufpi.br/xmlui/bitstream/handle/123456789/2057/defesa%2021%2012%202018-FINAL.pdf?sequence=1> (acessed 10 March, 2021)

31.	Cleveland RB, Cleveland WS, Mcrae JE, Terpenning I. STL: A Seasonal-Trend Decomposition Procedure Based on Loess. J Off Stat. 1990; 6(1):3-73. Available from: <https://www.wessa.net/download/stl.pdf> (acessed 10 March, 2021)

32.	Theodosiou M. “Forecasting monthly and quarterly time series using STL decomposition”. Int J Forecast. 2011; 27(4):1178-1195. Available from: <https://doi.org/10.1016/j.ijforecast.2010.11.002>

33.	Brockwell PJ, Davis RA. Introduction to time series and forecasting. New York: Springer-Verlag; 2002.

34.	Gómez, L., Rivera, A., Vidal, Y., Bilbao, J., Kasang, C., Parisi, S., Schwienhorst-Stich, E.-M. and Puchner, K.P: Factors associated with the delay of diagnosis of leprosy in north-eastern Colombia: a quantitative analysis. Trop Med Int Health; 2018; 23(2): 193-198. Available from: <https://doi.org/10.1111/tmi.13023>

35.	Henry M, Galan N, Teasdale K, Prado R, Amar H, Rays MS, et al. Factors contributing to the delay in diagnosis and continued transmission of leprosy in Brazil - an explorative, quantitative, questionnaire based study. PLoS Negl Trop Dis. 2016; 10:e0004542. Available from: <https://doi.org/10.1371/journal.pntd.0004542> PMID: 26977811

36.	Seshadri D, Khaitan BK, Khanna N, Sagar R. Dehabilitation in the era of elimination and rehabilitation: a study of 100 leprosy patients from a tertiary care hospital in India. Lepr Ver. 2015; 86:62-74. Available from: <https://pubmed.ncbi.nlm.nih.gov/26065148/> PMID: 26065148

37.	Price VG. Factors preventing early case detection for women affected by leprosy: a review of the literature. Glob Health Action. 2017 Jan-Dec;10(sup2):1360550. doi: 10.1080/16549716.2017.1360550.PMID: 28853325

38.	Barbosa DRM, Almeida MG, Santos AG. Características epidemiológicas e espaciais da hanseníase no Estado do Maranhão, Brasil, 2001-2012. Med (Ribeirao Preto. Online). 2014; 47(4):347-356. Available from: <https://doi.org/10.11606/issn.2176-7262.v47i4p347-356>

39.	Ramos ACV, Neto MS, Arroyo LH, Yamamura M, Assis IS, Alves JD, et al. Magnitude of social determinants in high risk areas of leprosy in a hyperendemic city of northeastern Brazil: An ecological study. Leprosy Rev. 2020; 91:41-55. Available from: <https://doi.org/10.47276/lr.91.1.41>

40.	Marciano LHSC, Belone AFF, Rosa PS, Coelho NMB, Ghidella CC, Nardi SMT, et al. Epidemiological and geographical characterization of leprosy in a Brazilian hyperendemic municipality. Cad Saude Publica. 2018; 34:e00197216. Available from: <https://doi.org/10.1590/0102-311x00197216>

41.	Almeida MG, et al. Saúde e masculinidade: uma calamidadenegligenciada. Anais do IV Congresso Internacional de Es-tudos sobre Diversidade Sexual e de Gênero da ABEH. 2012

42.	Oliveira MHP, Romanelli G. Os efeitos da hanseníase em homens e mulheres: um estudo de gênero. Cad. Saúde Pública  [Internet]. 1998;  14( 1 ): 51-60. Available from: <https://doi.org/10.1590/S0102-311X1998000100013>.

43.	Couto MT, G R. Men, health and public policies: gender equality in question. Ciênc. saúde coletiva  [Internet]. 2012;  17( 10 ): 2569-2578.   Available from: <http://dx.doi.org/10.1590/S1413-81232012001000002>.

44.	Moura EC, Gomes R, Pereira GMC. Perceptionsaboutmen’shealth in a genderrelational perspective, Brazil, 2014. Ciênc saúde coletiva. 2017; 22(1):291-300. Available from: <https://doi.org/10.1590/1413-81232017221.17482015>

45.	Lopes VAS, Rangel EM. Leprosy and social vulnerability: an analysis of the socioeconomic profile of users in irregular treatment. Saúde Debate. 2014; 38(103):817-829. Available from: <https://doi.org/10.5935/0103-1104.20140074>

46.	Souza EA, Ferreira AF, Boigny RN, Alencar CH, Heukelbach J, Martins-Melo FR, et al. Leprosy and gender in Brazil: trends in an endemic area of the Northeast region, 2001–2014. Rev Saude Publica. 2018; 52(20). Available from: <https://doi.org/10.11606/s1518-8787.2018052000335>

47.	Costa RSL, Pinto FASP, Silva MV. Reported cases of leprosy in the state of acre in the year of 2017. DêCiênc Foco. 2019; 3(2):15-25. Available from: <http://revistas.uninorteac.com.br/index.php/DeCienciaemFoco0/article/view/318/91> (acessed 10 March, 2021)

48.	Xavier MB, Tavares NCS, Corrêa SC, Gonçalves BK, Ramos MAB, Macedo GMM. Correlation between the clinical forms of leprosy and the degree of neurological incapacity. Rev Para Med. 2014; 28(2):15-21. Available from: <http://files.bvs.br/upload/S/0101-5907/2014/v28n2/a4253.pdf> (acessed 10 March, 2021)

49.	Balata ILB. Incapacidade física em menores de 15 anos com diagnóstico de hanseníase [Trabalho de conclusão de curso]. Universidade Federal do Maranhão, Centro de ciências biológicas e da saúde curso de enfermagem; 2018. Available from: <https://monografias.ufma.br/jspui/bitstream/123456789/2066/1/IngridBalata.pdf> (acessed 10 March, 2021)

50.	Freitas BHBM, Cortela DCB, Ferreira SMB. Trend of leprosy in individuals under the age of 15 in Mato Grosso (Brazil), 2001-2013. Rev Saúde Pública. 2017; 51:28. Available from: <http://dx.doi.org/10.1590/s1518-8787.2017051006884>

51.	Ribeiro MDA, Silva JCA, Oliveira SB. Epidemiologic study of leprosy in Brazil: reflections on elimination goals. Rev Panam Salud Publica. 2018; 42(1):e42. <https://doi.org/10.26633/RPSP.2018.42>

52.	Croft RP, Nicholls PG, Steyerberg EW, Richardus JH, Cairns W, Smith S. A clinical prediction rule for nerve-function impairment in leprosy patients. Lancet. 2000; 355(9215):1603-1606. Available from: <https://doi.org/10.1016/s0140-6736(00)02216-9> PMID: 10821364

53.	Gomes KKL, Calado AMC. Análise do modo de detecção da hanseníase na Paraíba (2006-2016). Anais VI CONGREFIP. Campina Grande: Realize Editora; 2017 Available from: <https://editorarealize.com.br/artigo/visualizar/27744> (acessed 10 March, 2021)

54.	Monteiro LD, Alencar CHM, Barbosa JC, Braga KP, Castro MD, Heukelbach J. Physical disabilities in leprosy patients after discharge from multidrug therapy in Northern Brazil. Cad Saude Publica. 2013; 29(5):909–920. Available from: <https://doi.org/10.1590/S0102-311X2013000500009>

55.	Alves ACR, Lemos GS, Paiva PDR. Perfil socioeconômico dos pacientes atendidos pelo Centro de Referência em Reabilitação da Hanseníase da Zona da Mata Mineira. HU Rev. 2017; 43(2):99-104. Available from: <https://doi.org/10.34019/1982-8047.2017.v43.2640>

56.	Pereira TM, Silva LMS, Dias MAS, Monteiro LD, Silva MRF, Alencar OM. Temporal trend of leprosy in a region of high endemicity in the Brazilian Northeast. Rev Bras Enferm. 2019; 72(5):1356-1362. Available from: <https://doi.org/10.1590/0034-7167-2018-0682>

57.	Pinheiro HHC. Análise de série temporal da hanseníase no estado do Pará [Tese]. Universidade Federal do Pará, Núcleo de Medicina Tropical, Programa de Pós-Graduação em Doenças Tropicais; 2016. Available from: <http://repositorio.ufpa.br/jspui/bitstream/2011/9108/1/Tese_AnaliseSerieTemporal.pdf> (acessed 10 March, 2021)

58.	Penna ML, Pedrosa VL, Pereira ES. Leprosy decline in Amazonas State Brazil. Trop Med Int Health. 2012; 17(2):244-246. Available from: <https://doi.org/10.1111/j.1365-3156.2011.02900.x> PMID: 22035216

59.	Moet FJ, Pahan D, Oskam L, Richardus JH; COLEP Study Group. Effectiveness of single dose rifampicin in preventing leprosy in close contacts of patients with newly diagnosed leprosy: cluster randomized controlled trial. BMJ. 2008;336(7647):761–4. doi: 10.1136/bmj.39500.885752.BE.

**Supporting information**

**S1 File. STROBE Statement—checklist of items that should be included in reports of observational studies.**

**S2 Dataset. Minimal anonymized data set.**

**S3 Fig. 2 Trends in total detection rates, in the general population, by gender and age groups per 100,000 inhabitants in Cuiabá (2008–2018). (Black line) Time series; (Red line) Trend.**

**S4 Fig. 4 Trends in the gross number of cases of Grade of Disability at Diagnosis (DPD) in patients diagnosed with leprosy in the period from 2008 to 2018 in Cuiabá. (Black line) Time series; (Red line) Trend.**

**S5 Fig. 5 Trends in the gross number of cases of Grade of Disability at Diagnosis (DPD) by gender in patients diagnosed with leprosy in the period from 2008 to 2018 in Cuiabá. (Black line) Time series; (Red line) Trend.**

**S6 Fig. 6 . Trends in the gross number of cases of Grade of Disability at Diagnosis (DPD) by age group in patients diagnosed with leprosy in the period from 2008 to 2018 in Cuiabá. (Black line) Time series; (Red line) Trend.**
