# Supplementary material for: Inequality of gender, age and disabilities due to leprosy and trends in a hyperendemic metropolis: Evidence from an eleven-year time series study in Central-West Brazil
Source: PLoS Negl Trop Dis. 2021 Nov 16;15(11):e0009941. doi: 10.1371/journal.pntd.0009941 (PMC8631739; doi:10.1371/journal.pntd.0009941)
Supplement: Attachment — Submitted filename: Response to Reviewers.docx [file pntd.0009941.s009.docx]

Dear Editor,

Many thanks for your reply and your reviewers' comments about our manuscript “PNTD-D-21-00361R1 Inequality of gender, age and disabilities due to leprosy and trend in a hyperendemic metropolis: Evidence from an eleven-year time series study in Central-West Brazil”.

The comments were appropriate to qualify/ improve the manuscript. We have forwarded a letter with the changes made in the manuscript, presenting our response for each comment from reviewers. Many thanks for your comments.

The authors tried their best to reduce the discussion and introduction also tried to improve the English language and explanation of the most important aspects of the manuscript. All the suggestions made in the file attached including the comments were accepted and included in the manuscript.

Below, we respond point by point to the comments of all Reviewers.

Kind regards,

Martoreli Júnior et al.

**Reviewers comments:**

**Reviewer #2:**

**TITLE**

**"hyperendemic" is written with space in the rest of the manuscript, but without in the title**

**Authors:** Corrected in the entire manuscript using the following reference:

11.	Ramos ACV, Gomes D, Neto MS, Berra TZ, Assis IS, Yamamura M, et al. Trends and forecasts of leprosy for a hyperendemic city from Brazil’s northeast: Evidence from an eleven-year time-series analysis. PLoS One. 2020; 15(8):e0237165. Available from: <http://dx.doi.org/10.1371/journal.pone.0237165> PMID: 32764785

**INTRODUCTION**

**Most changes made were appreciated.- See my suggested changes and comments in the text file attached.**

**Authors:** Accepted and included all suggestions and comments in the text file attached.

**Please include the latest version oif the WHO Global Leprosy Strategy (2021-2030).**

**Authors:** We updated the version of the WHO Global Strategy by the present year (2021-2030).

**I would delete: "Also, regarding the inequality related to age, it is known that when there is a delay in diagnosis, children that who had contact with index cases can also become ill [1112], which is an important gap to be filled."**

**Authors:** Deleted.

**METHODS**

**Most changes made were appreciated. See my suggested changes and comments in the text file attached.**

**Authors:** Accepted and included all suggestions and comments in the text file attached.

**You could think of adding a definition table with an overview of the disability grades (what is G0D, G1D, G2D)**

**Authors:** We’ve add a definition table in the line 197 based on the reference 26:

22. MINISTÉRIO DA SAÚDE, Roteiro para uso do Sistema de Informação de Agravos de Notificação – Sinan NET para hanseníase 2018. Available from: <http://portalsinan.saude.gov.br/images/documentos/Agravos/Hanseniase/Manual_tabulacao_dos_indicadores_de_hanseniase.pdf>

**Capital usage still not fully correct**

**Authors:** The Authors tried to correct the Capital usage in the entire manuscript.

**- This sentence is unclear: "Regarding  the disability gradethe grade of disability, health units evaluated a mean of 56.24% of new cases with a disability at the time of diagnosis, below expectations, which was listsed as should be at least 75.00% according to SINAN  [235, 26]."**

**Authors:** We rewritten the sentence in lines 167 - 173:

“According to Ministry of Health on the SINAN document for the data analysis of leprosy in Brazil, at least 75.00% of new cases on the time of the diagnoses need evaluation for the grade of disability [25,26]. On the Official Epidemiologic Bulletin of Cuiabá of the years 2017, 2018 and 2019 health units evaluated a mean of 56.24% of new cases at the time of diagnosis, below expectations, which should be at least 75.00% according to SINAN [25, 26].”

21.	Brasil. Prefeitura de Cuiabá. Boletim Epidemiológico Hanseníase. Cuiabá: SES; 2019. Available from: <http://201.24.3.67:8080/portal/upload/arquivos/20190523165956000163.pdf> (acessed 10 March, 2021)

22.	MINISTÉRIO DA SAÚDE, Roteiro para uso do Sistema de Informação de Agravos de Notificação – Sinan NET para hanseníase 2018. Available from: <http://portalsinan.saude.gov.br/images/documentos/Agravos/Hanseniase/Manual_tabulacao_dos_indicadores_de_hanseniase.pdf>

**- I donnot understand this sentence because of the level of English, you can maybe also delete it: "For the construction of time series of the cases with disability grade we considered all the number of cases with disability grade (G0D, G1D, G2D, and not evaluated)."**

**Authors:** Deleted.

**- This is also quite vague, could it be deleted? "... even for a very long time series and large amounts of trend and seasonal smoothing, a very small amount of trend smoothing, and seasonal components that are not distorted by aberrant behavior in the data ability to decompose time series with missing values"**

**Authors:** Deleted.

**- This should not be in the methods section, but in the discussion (is already included there). Also, the word 'dubious' is too strong/incorrect: "Finally, we mention that the notification forms are filled out by third parties, so dubious data may be provided."**

**Authors:** Deleted in the methods section.

**'ignored' is too strong in table 1, go for: 'not classified / incorrect / incomplete' instead**

**Authors:** Accepted the suggestion and changed by incomplete.

**DISCUSSION**

**Most changes made were appreciated.See my suggested changes and comments in the text file attached.**

**Authors:** Accepted all suggestions and comments in the text file attached.

**Improvements can be made regarding: item order in the discussion, repetition, level of English.**

**Authors:** The authors have updated the discussion, reorganized, and make it clearer considering the suggestion from reviewer. We informed that the manuscript has been reviewed by Proof-Reading-Service.com, which is specialized and offers professional journal article editing.

**- I would suggest to delete this, as it seems less relevant (not clearly explained how this affects the leprosy programme) and the discussion word count is too high: "In the state of Mato Grosso, in 2015, the “National Campaign for Leprosy, Geohelminthiasis and Trachoma” was initiated, which mobilized local health services to execute carry out actions related to the active search for cases, focusing on schoolchildren, aged from 5 to 14 years. The campaign was carried out in approximately 915 schools in 65 municipalities (including Cuiabá), to examine and treat more than 291..200 thousand students and possibly their possible contacts [242]. It is estimated that the campaign may have had an impact in the region studied, reflecting the peak of detection verified in the study for this age group. No further policies were encountered in public archives to influence the detection of leprosy in the region duringh the time period."**

**Authors:** The authors appreciate the comment but choose to keep the paragraph. The campaign “National Campaign for Leprosy, Geohelminthiasis and Trachoma” was a significant national campaign for the detection of leprosy in Brazil and in the studied setting. The analyzes showed that specifically in 2015, the year the campaign started, there was a significant increase in the detection of cases shown in Fig 2 (S3_Figure2).

**CONCLUSION**

**- See my suggested changes and comments in the text file attached.**

**Authors:** Accepted all suggestions in the text file attached.

**Improvements were made and are much appreciated. Nevertheless, the manuscript still need to be revised. Especially the discussion needs attention, the text order should be changed and repetition should be avoided. Both the introduction and discussion section can be shorter and more to the point. The newly written text segments need improvents regarding the level of English.**

**Authors:** The authors appreciate the comment and have tried to reduce the discussion and introduction.

**Reviewer #4:**

**See attachment**

**Authors:** The authors are grateful for the comments and accepted all suggestions and comments made in the file attached.

**I have made some edits to the text (attached, visible with track changes). That is easier for me than repeating everything in this form one by one.**

**Authors:** The authors are grateful for the comments and accepted all suggestions.

**The paper has improved well after a thorough revision. There are still some small issues that I have addressed directly in the revised manuscript with track changes visible. This is not intended as a full language edit, but just to improve on some relatively important matters with regard to language and explanation.**

**Authors:** The authors are grateful for the comment and have tried to improve the English language and explanation of the most important aspects of the manuscript.
