# Supplementary material for: Inequality of gender, age and disabilities due to leprosy and trends in a hyperendemic metropolis: Evidence from an eleven-year time series study in Central-West Brazil
Source: PLoS Negl Trop Dis. 2021 Nov 16;15(11):e0009941. doi: 10.1371/journal.pntd.0009941 (PMC8631739; doi:10.1371/journal.pntd.0009941)
Supplement: Attachment — Submitted filename: Response to Reviewers.docx [file pntd.0009941.s011.docx]

Dear Editor,

Many thanks for your reply and your reviewers comments about our manuscript “PNTD-D-21-00361R2 Inequality of gender, age and disabilities due to leprosy and trend in a hyperendemic metropolis: Evidence from an eleven-year time series study in Central-West Brazil”.

The comments were appropriate to qualify/ improve the manuscript. We have forwarded a letter with the changes made in the manuscript, presenting our response for each comment from reviewers and also a response for the comments made in the Word file. Many thanks for your comments.

The authors tried their best to reduce the methods section and make it easier to read. The suggestions made in the file attached including the comments were included in the manuscript. The references section was checked to ensure that it was compatible with the excerpts cited by them.

Only the orders of a few paragraphs of the results were changed so that the figures are presented in an increasing numerical form.

Below, we respond point by point to the comments of the Reviewers.

Kind regards,

Martoreli Júnior et al.

**Reviewers comments:**

**Reviewer #2:**

**The methods section can still be shortened, see if you can delete some sentences, this will make it easier to read.
Authors:** The authors tried to reduce as much as possible without compromising the understanding of the manuscript.

**I suggested in the Word document to move a text section from the 'Methods' to the 'Discussion'.**
**Authors:** Moved.

**Please look at my other suggested changes (tracked changes & yellow marked) and comments in the Word file.:**

**I added this as this is the preferred name in Brazil, and when including the word in the abstract, it will be more easily found via search engines like PubMed**

**Authors:** The authors were grateful for the contribution and accepted the addition.

**This sentence is not reading well in English. Also, it is too detailed for the abstract. I would delete the second half of sentence and only keep: "The calendar adjustment technique was applied."**

**Authors:** Deleted.

**G2D % is more relevant, please add**

**Authors:** The autors add the date about G2D in the author summary.

**You are using the wrong reference here, this should be: WHO. Towards zero leprosy. Global leprosy (‎Hansen’s Disease)‎ strategy 2021–2030. April 2021. https://www.who.int/publications/i/item/9789290228509 (accessed ….).**

**Authors:** We modified as requested. All references were reviewed and compared with the excerpts cited by them.

**So, in this sentence, you are referring to 2 WHO documents (the Guidelines & the Strategy), and thus should include both references:**

**WHO 2018. Guidelines for the Diagnosis, Treatment and Prevention of Leprosy. Geneva: World Health Organization; 2018. https://apps.who.int/iris/handle/10665/274127 (accessed 10 March, 2021).**

**Authors:** This will be reference 7.

**WHO. Towards zero leprosy. Global leprosy (‎Hansen’s Disease)‎ strategy 2021–2030. April 2021. https://www.who.int/publications/i/item/9789290228509 (accessed ….).**

**Authors:** This will be reference 1 and we updated the excerpts according to this new reference.

**Add reference(s) for these hypotheses**

**Authors:** We add the following references:

10.	Oliveira JSS, Reis ALMD, Margalho LP, Lopes GL, Silva ARD, Moraes NS, et al. Leprosy in elderly people and the profile of a retrospective cohort in an endemic region of the Brazilian Amazon. PLoS Negl Trop Dis. 2019; 13(9):e0007709. Available from: http://dx.doi.org/10.1371/journal.pntd.0007709 PMID: 31479442

11.	Vieira MCA, Nery JS, Paixão ES, Andrade KVF, Penna GO, Teixeira MG. Leprosy in children under 15 years of age in Brazil: A systematic review of the literature. PLoS Negl Trop Dis. 2018; 12(10):e0006788. Available from: http://dx.doi.org/10.1371/journal.pntd.0006788 PMID: 30278054

**The word "behaviour" should only be used for human beings and animals. Please use e.g. "trend" here**.

**Authors:** Changed as requested.

**You may like to include the full official names here besides the abbreviations, e.g. : “dermatology surveillance service (CERMAC, …full name in Portuguese….)”**

**Authors:** Included the full official names in Portuguese:

“…CERMAC - (dermatology surveillance services) (centro estadual regional de média e alta complexidade), metropolitano hospital (hospital care for leprosy surgery services), Julio Muller University Hospital (hospital care for ophthalmology referral), CRIDAC center specialized in rehabilitation (CER III) (centro de reabilitação integral dom aquino corrêa) , and regional outpatient and hospital referrals…”

**Which category of by whom?**

**Authors:** Health Surveillance Department of Brazil. Ministry of health, Health Surveillance Department, Department of Epidemiological Surveillance of Communicable Diseases, General Coordination of Leprosy and Elimination Diseases, Page 53:

“…Annual detection rate of new leprosy cases, in a population aged zero to 14 years, per 100,000 inhab.

Very high 5,00 a 9,99/100.000 hab...”

23.	Ministério da saúde , Roteiro para uso do Sistema de Informação de Agravos de Notificação – Sinan NET para hanseníase 2018. Available from: http://portalsinan.saude.gov.br/images/documentos/Agravos/Hanseniase/Manual_tabulacao_dos_indicadores_de_hanseniase.pdf

**Unclear, also make this consistent (writing Fig or Figure) throughout the whole document**

**Authors:** Changed all by Fig. The names are the same in the Supporting information.

**People without leprosy ? / What is meant here? Leprosy patients with disabilities compared to all leprosy patients, or compared to the normal (non-leprosy) popilation?**

**Author:** The authors corrected the subtitles of the tables to make them clear.

**Delete this, you are always the source if it is in your article and if you don’t add another reference**

**Authors:** Deleted.

**You could change the term ‘behavior’ into ‘pattern’. The word behavior is normally associated with living beings, not figures.**

**Authors:** Deleted the term.

**LPEP is the name of the study, not the name of the medicine: PEP**

**Authors:** Corrected.

**Reference is incorrect, this should be: Moet FJ, Pahan D, Oskam L, Richardus JH; COLEP Study Group. Effectiveness of single dose rifampicin in preventing leprosy in close contacts of patients with newly diagnosed leprosy: cluster randomized controlled trial. BMJ. 2008;336(7647):761–4. doi: 10.1136/bmj.39500.885752.BE.**

**Authors:** This is the reference 34 now.

34	Moet FJ, Pahan D, Oskam L, Richardus JH; COLEP Study Group. Effectiveness of single dose rifampicin in preventing leprosy in close contacts of patients with newly diagnosed leprosy: cluster randomized controlled trial. BMJ. 2008;336(7647):761–4. doi: 10.1136/bmj.39500.885752.BE.

**A mean of what, G2D registrations? /All should be evaluated for disability**

**Authors:** Rewritten the phrases and add the suggestion:

“According to the Ministry of Health quality control, at least 75.00% of new cases at the time of diagnosis need to be evaluated for the disability grade and registered into SINAN [23, 24]. In the Official Epidemiologic Bulletin of Cuiabá of the years 2017, 2018 and 2019 health units evaluated 56.24% G2D registration of new cases at the time of diagnosis, below expectations [22]. The authors of this study emphasize that a 100% of disability grade assessment and registration at time of diagnosis, as well as for disease progression evaluation, should be pursued.”

22.	Brasil government. Prefeitura de Cuiabá. Boletim Epidemiológico Hanseníase. Cuiabá: SES; 2019. Available from: http://201.24.3.67:8080/portal/upload/arquivos/20190523165956000163.pdf (acessed 10 March, 2021)

23.	Ministério da saúde , Roteiro para uso do Sistema de Informação de Agravos de Notificação – Sinan NET para hanseníase 2018. Available from: http://portalsinan.saude.gov.br/images/documentos/Agravos/Hanseniase/Manual_tabulacao_dos_indicadores_de_hanseniase.pdf

24.	Ministério da saúde , Guia prático sobre a Hanseníase – Secretaria de Vigilância em Saúde – Departamento de Vigilância e Doenças Transmissíveis - Brasília - DF 2017 Availble from: https://portalarquivos2.saude.gov.br/images/pdf/2017/novembro/22/Guia-Pratico-de-Hanseniase-WEB.pdf

**This is what you mean here (pb, mb, etc?), correct?**

**Authors:** Yes.

**Besides the WHO Guidelines & Moet et al, it would be best to also add the (re-)references below (some you already used elsewhere) :**

**Richardus, J. H., Tiwari, A., Barth-Jaeggi, T., Arif, M. A., Banstola, N. L., Baskota, R., ... & Steinmann, P. (2021). Leprosy post-exposure prophylaxis with single-dose rifampicin (LPEP): an international feasibility programme. The Lancet Global Health, 9(1), e81-e90. https://www.sciencedirect.com/science/article/pii/S2214109X2030396X**

**Authors:** Reference 54.

54.	Richardus, J. H., Tiwari, A., Barth-Jaeggi, T., Arif, M. A., Banstola, N. L., Baskota, R., ... & Steinmann, P. (2021). Leprosy post-exposure prophylaxis with single-dose rifampicin (LPEP): an international feasibility programme. The Lancet Global Health, 9(1), e81-e90. https://www.sciencedirect.com/science/article/pii/S2214109X2030396X

**WHO. Leprosy/Hansen disease: Contact tracing and post-exposure prophylaxis. November 2020. https://www.who.int/publications/i/item/9789290228073**

**Authors:** Reference 55

55.	WHO. Leprosy/Hansen disease: Contact tracing and post-exposure prophylaxis. November 2020. https://www.who.int/publications/i/item/9789290228073

**WHO. Towards zero leprosy. Global leprosy (‎Hansen’s Disease)‎ strategy 2021–2030. April 2021.** [**https://www.who.int/publications/i/item/9789290228509**](https://www.who.int/publications/i/item/9789290228509)

**Authors:** Reference 1

1.	WHO 2021 Towards zero leprosy. Global leprosy (‎Hansen’s Disease)‎ strategy 2021–2030; 2021, World Health Organization – Licence: CC BY-NC-SA 3.0 IGO., ISBN: 978 92 9022 850 9. Available from: https://www.who.int/publications/i/item/9789290228509

**First time this abbreviation is used, please use the full wording**

**Authors**: It was already used in the Statistic analysis section:

“Having estimated the three components of the time series, only the trend was selected to characterize the trend of the variables of interest over time. Subsequently, the Average Monthly Percentage Change (AMPC) was calculated…”.

**This should be in the Discussion section**

**Authors:** Moved to the Discussion section:

“In the data, we encountered some operational classification with incompatibility with clinical form, such as ‘indeterminate’ being classified as ’multibacillary’ cases, thus having to add ’not classified/incomplete’ on operational classification. Not all clinical forms were filled in the databank contrary to the operational classification, causing disparities between the sum of them.”

**If possible, further shorten the 'Methods'-section.**

**Authors:** The authors tried their best to shorten this section without removing the information that makes the reader understand the location of the study.

**Describe where the raw data can be found for reproducibility reasons (see my comment/addition in 'Ethics'-section).**

After approval by the ethics committee of the University of são Paulo, secondary data were collected and sent to the authors. The anonymized data were sent through an Excel document to the Journal as an Supporting information with the name S2_Dataset.xlsx.
 **Please check the references, some are incorrect in the text and inconsistent in the reference list.**

**Authors:** The references section was checked to ensure that it was compatible with the excerpts cited by them.
